# Identification of Antioxidant Methyl Derivatives of *Ortho*-Carbonyl Hydroquinones That Reduce Caco-2 Cell Energetic Metabolism and Alpha-Glucosidase Activity

**DOI:** 10.3390/ijms25158334

**Published:** 2024-07-30

**Authors:** Matías Monroy-Cárdenas, Cristopher Almarza, Paulina Valenzuela-Hormazábal, David Ramírez, Félix A. Urra, Maximiliano Martínez-Cifuentes, Ramiro Araya-Maturana

**Affiliations:** 1 Escuela de Química, Facultad de Química y de Farmacia, Pontificia Universidad Católica de Chile, Av. Vicuña Mackenna 4860, Santiago 7820436, Chile; 2 MIBI—Interdisciplinary Group on Mitochondrial Targeting and Bioenergetics, Universidad de Talca, P.O. Box 747, Talca 3460000, Chile; c.chavez.bio@gmail.com (C.A.); felixurraf@uchile.cl (F.A.U.); 3 Network for Snake Venom Research and Drug Discovery, Av. Independencia 1027, Santiago 7810000, Chile; 4 Metabolic Plasticity and Bioenergetics Laboratory, Molecular and Clinical Pharmacology Program, Institute of Biomedical Sciences, Faculty of Medicine, University of Chile, Av. Independencia 1027, Santiago 7810000, Chile; 5 Departamento de Farmacología, Facultad de Ciencias Biológicas, Universidad de Concepción, Concepción 4030000, Chile; paulinvalenzuela@udec.cl (P.V.-H.); dramirezs@udec.cl (D.R.); 6 Departamento de Química Orgánica, Facultad de Ciencias Químicas, Universidad de Concepción, Edmundo Larenas 129, Concepción 4070371, Chile; 7 Instituto de Química de Recursos Naturales, Universidad de Talca, Talca 3460000, Chile

**Keywords:** hydroquinones, methyl derivatives, antioxidants, diabetes, DFT, docking

## Abstract

α-glucosidase, a pharmacological target for type 2 diabetes mellitus (T2DM), is present in the intestinal brush border membrane and catalyzes the hydrolysis of sugar linkages during carbohydrate digestion. Since α-glucosidase inhibitors (AGIs) modulate intestinal metabolism, they may influence oxidative stress and glycolysis inhibition, potentially addressing intestinal dysfunction associated with T2DM. Herein, we report on a study of an *ortho*-carbonyl substituted hydroquinone series, whose members differ only in the number and position of methyl groups on a common scaffold, on radical-scavenging activities (ORAC assay) and correlate them with some parameters obtained by density functional theory (DFT) analysis. These compounds’ effect on enzymatic activity, their molecular modeling on α-glucosidase, and their impact on the mitochondrial respiration and glycolysis of the intestinal Caco-2 cell line were evaluated. Three groups of compounds, according their effects on the Caco-2 cells metabolism, were characterized: group A (compounds **2**, **3**, **5**, **8**, **9**, and **10**) reduces the glycolysis, group B (compounds **1** and **6**) reduces the basal mitochondrial oxygen consumption rate (OCR) and increases the extracellular acidification rate (ECAR), suggesting that it induces a metabolic remodeling toward glycolysis, and group C (compounds **4** and **7**) increases the glycolysis lacking effect on OCR. Compounds **5** and **10** were more potent as α-glucosidase inhibitors (AGIs) than acarbose, a well-known AGI with clinical use. Moreover, compound **5** was an OCR/ECAR inhibitor, and compound **10** was a dual agent, increasing the proton leak-driven OCR and inhibiting the maximal electron transport flux. Additionally, menadione-induced ROS production was prevented by compound **5** in Caco-2 cells. These results reveal that slight structural variations in a hydroquinone scaffold led to diverse antioxidant capability, α-glucosidase inhibition, and the regulation of mitochondrial bioenergetics in Caco-2 cells, which may be useful in the design of new drugs for T2DM and metabolic syndrome.

## 1. Introduction

Diabetes mellitus (DM) is a metabolic chronic disease, highly prevalent in both the developing and developed world [1]. It is characterized by inadequate glycemic control, caused by either reduced insulin secretion (type 1 diabetes mellitus, T1DM) or by a reduced cellular response to insulin (type 2 diabetes mellitus, T2DM) [2,3,4], which is the main type of diabetes, with a prevalence of the 90–95% of the total incidence [5]. In T2DM, enhanced ROS production via reduced intracellular antioxidant levels is observed [6,7], which, at the same time, accelerates the dysregulation of glucose metabolism and tissue damage through signal transductions.

Molecular crosstalk between microRNAs and oxidative stress in the pathogenesis of T2DM and its related health problems has been pointed out [6]. Also, recently, it has been suggested that miRNAs and oxidative stress influence each other. Oxidative stress seems to regulate the function and biogenesis of several miRNAs and vice versa. An imbalance in miRNA expression can lead to oxidative stress by promoting free radical generation and (or) reducing the endogenous antioxidant capacity [8,9,10].

On the other hand, α-glucosidase is an emerging target in developing novel T2DM agents such as PTP1B and aldose reductase, among others [11,12,13]. α-glucosidase is present in the brush border membrane of intestinal cells [14]. It catalyzes the hydrolysis of the α-(1,4)-glycosidic linkage of sugar (disaccharides and starch), releasing free monosaccharides (α-D-glucose) during the final step of carbohydrate digestion. The α-glucosidase inhibitors (AGIs) can suppress postprandial hyperglycemia and decrease carbohydrate digestion rate, therefore reducing the glucose level in the bloodstream [15,16,17,18,19,20,21].

It is known that dietary polyphenols modulate post-prandial increases in glucose levels, intestinal integrity, and oxidative damage [22,23,24]. Consequently, a growing interest in this type of compound has been observed in the last few years. It has been suggested that oxidative energy metabolism plays a crucial role in mitochondrial ATP production and in maintaining a gut barrier with high integrity [25]. The disruption of this intestinal barrier function is related to inflammation [26] and dysmetabolic conditions, including T2DM [27]. In this context, the human intestinal epithelial Caco-2 cell line has been used to assess the relationship between mitochondrial ATP production and intestinal permeability [28].

Previously, we have reported an *ortho*-carbonyl hydroquinone series that disrupts mitochondrial bioenergetics in cancer cells by Complex I inhibition and oxidative phosphorylation uncoupling without affecting non-cancer cells [29,30,31]. On the other hand, it has been described that certain hydroquinones and quinones can induce redox cycling [32,33,34,35]; however, this is not the case with these hydroquinones [30]. Since they exhibit a high antioxidant capability in cell-free analysis [36] and, as has been discussed above, oxidative stress is involved in the intestinal dysfunction associated with T2DM, this work aims to study the antioxidant capacity of the methyl derivatives of a bicyclic *ortho*-carbonyl hydroquinone (Table 1), which differs only in the number and position of methyl groups, and its effects as an AGI, and in modulating the energetic metabolism of Caco-2 cells.

## 2. Results

### 2.1. Synthesis of the Compounds

**Scheme 1:**
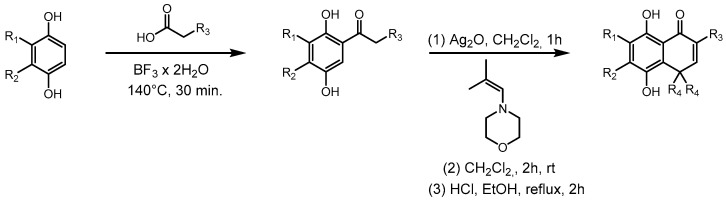
Synthesis of methyl derivatives of *ortho*-carbonyl hydroquinones. R corresponds to those shown in Table 1.

The new hydroquinones (Scheme 1) were obtained in two steps. The first consists of obtaining acylhydroquinones following the reported method [37,38] with a slight modification. To a Reaction Vial G10 (Anton-Paar, Graz, Austria) equipped with a stir bar, the following were added: 500 mg of corresponding hydroquinone, a 1.5 equivalent of the corresponding carboxylic acid, and 3 mL of Boron trifluoride dihydrate. The mixture was heated at 160 °C for 30 min in a Monowave 50 reactor (Anton-Paar). The mixture was allowed to cool to room temperature, neutralized with saturated sodium bicarbonate solution, and extracted with ethyl acetate. The extract was washed with distilled water, dried with anhydrous sodium sulfate, and then filtered and concentrated in a rotavapor. Afterward, the corresponding acylhydroquinone was purified by flash chromatography with hexane/ethyl acetate 8:1 *v*/*v* as an eluent. The second step was performed following reference [39], although this was also done with slight variations. A mixture of corresponding acylhydroquinone (1 equiv) and Ag_2_O (2.5 equiv) in 30 mL of dichloromethane was vigorously stirred for 1–2 h at r.t., yielding the corresponding quinone. This mixture was filtered through celite and added dropwise to a solution of the 4- (2-methyl-2-propenyl)morpholine or 4-(2-ethylbut-1-en-1-yl)morpholine at r.t. for 2 h, being monitored by thin-layer chromatography. Then, the solvent was evaporated under reduced pressure, and the residue was dissolved in a mixture of ethanol and hydrochloric acid and refluxed by 1 h. Next, it was poured on an ice/water mixture, and the product was extracted with 3 portions of 20 mL of ethyl acetate, which were dried with anhydrous sodium sulfate and then filtered; the solvent was evaporated under reduced pressure and purified by flash chromatography using hexane/ethyl acetate 4:1 *v*/*v* as an eluent.

### 2.2. In Vitro Antioxidant Capacity

The radical-scavenging activities of the hydroquinone series against peroxyl radicals were assessed (ORAC assays). The results are presented in Table 2. For the series with the *gem*-dimethyl on ring B (compounds **1**–**5**), compound **1** showed the highest antioxidant capacity (8.8 + 0.2 mol TE/mol comp). Similarly, for the series with the *gem*-diethyl group on ring B (compounds **6**–**10**), compound **6** presents the highest antioxidant capacity (7.0 + 0.3 mol TE/mol compound). These results indicate that introducing methyl substituents such as R1, R2, and R3 decreases the antioxidant capacity of the compounds. The replacement of the *gem*-dimethyl with the *gem*-diethyl group also decreases the antioxidant capacity.

### 2.3. Calculation of Main Thermodynamic Parameters

To obtain insight into the mechanism responsible for the antioxidant capacity of the compounds, we carried out DFT calculations. We considered the three mechanisms commonly associated with radical scavenging [40]: Hydrogen atom transfer (HAT), sequential electron transfer-proton transfer (SET-PT), and sequential proton loss-electron transfer (SPLET). These mechanisms can occur in parallel but at different rates. The thermodynamic parameters associated with each mechanism can help to assess their feasibility. The HAT mechanism is associated with the bond dissociation enthalpy (BDE) [40]. The SET-PT mechanism is associated with ionization potential (IP) and proton dissociation enthalpy (PDE) [40]. The SPLET mechanism is associated with proton affinity (PA) and electron transfer enthalpy (ETE) [40]. We evaluated the BDE for both OH groups (1 and 2 in Table 2) present in all compounds (BDE 1 and BDE 2 in Table 2). The highest values correspond to BDE 1, suggesting that the intramolecular hydrogen bond (IAHB) C-H∙∙∙O impedes O-H dissociation. The BDEs of the O-H not involved in an IAHB presented values around 70 kcal/mol. Based on the above, the SET-PT and SPLET mechanisms only were evaluated for the estimation of PDE and PA for OH2.

**Table 2:** In vitro radical-scavenging activities (ORAC), calculated BDEs, IP, PDE, PA, and ETE (values in kcal/mol), and inhibitory effect of *ortho*-carbonyl hydroquinones on α-glucosidase activity. Data are shown as means ± SD of three independent experiments. * Calculated BDE of the O-H 1 for anions deprotonated at O-H 2. n.d.: not determined. ^n^: not significant, ^a^: *p* < 0.001 versus acarbose.

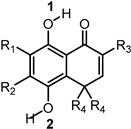									
Compound	ORAC	HAT		SET-PT		SPLET			Inhibition of α-Glucosidase Activity
	(mol TE/mol Compound)	BDE 1	BDE 2	IP	PDE 2	PA 2	ETE 2	BDE OH1 *	IC50 (µM)
**1**	8.8 + 0.2	90.07	71.96	178.08	207.21	329.02	56.28	65.09	190 + 2.2 ^n^
**2**	5.5 + 0.2	89.31	69.32	175.33	207.32	326.62	56.03	65.43	140 + 2.0 ^a^
**3**	3.9 + 0.1	87.62	70.92	173.67	210.58	329.52	54.73	63.93	>500
**4**	1.05 + 0.03	87.32	68.35	169.98	211.71	327.81	53.87	63.66	245 + 10.1 ^a^
**5**	0.37 + 0.01	86.78	67.95	168.50	212.78	328.83	52.45	63.72	103 + 2.9 ^a^
**6**	7.0 + 0.3	89.16	70.61	176.68	207.26	327.51	56.43	64.93	195 + 2.3 ^n^
**7**	7.2 + 0.4	89.58	69.26	173.95	208.64	326.81	55.77	65.75	110 + 1.6 ^a^
**8**	5.9 + 0.3	87.49	70.75	172.72	211.36	328.95	55.13	64.03	60 + 1.5 ^a^
**9**	0.83 + 0.03	86.89	68.70	169.18	212.85	327.25	54.78	64.08	>500
**10**	0.10 + 0.01	86.78	67.95	168.50	212.78	328.83	52.46	63.73	78 + 1.1 ^a^
Acarbose	n.d.	n.d.	n.d.	n.d.	n.d.	n.d.	n.d.	n.d.	194 + 1.7

**Figure 1:**
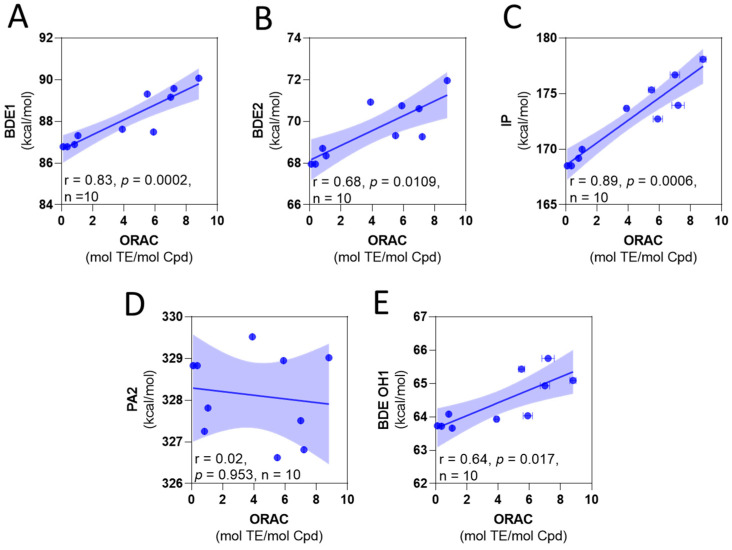
Correlation among calculated thermodynamic parameters and experimental ORAC values. (**A**) BDE of OH1 versus ORAC. (**B**) BDE of OH2 versus ORAC. (**C**) IP versus ORAC. (**D**) PA of OH2 versus ORAC. (**E**) BDE of OH1 for the anions deprotonated at OH2 versus ORAC.

To correlate to the experimental antioxidant capacity mechanism in relation to thermodynamic parameters, two approaches can be employed [41,42,43]. Some reports consider the value of the parameter as the key factor associated with a mechanism [41,42]; namely, the lower value, the higher their contribution of the mechanism. However, an alternative method is to consider the correlation between the experimental values of assays and thermodynamic parameters [43]. Following the first approach, the most favorable mechanism corresponds to the HAT, with the H-atom abstraction corresponding to the hydroxyl 2. Looking into the correlation between ORAC and the thermodynamic parameters (Figure 1), we found that the best correlation corresponds to the IP. However, the BDEs and IP slopes are positive (Figure 1A–C), indicating that high values of the thermodynamic parameter (theoretically a less potent antioxidant) correspond to the high values of ORAC (experimentally stronger antioxidants).

We also consider the hydroquinone anions, with the compounds deprotonated at hydroxyl 2, as contributing antioxidant species. The BDE for the O-H 1, obtained in this form, is presented in Table 2. These values were lower than those of neutral species. However, the correlation exhibited the same tendency regarding the experimental ORAC values (Figure 1D,E). These results suggest that the variation of the antioxidant capacity of the compounds is influenced by a factor not considered in the calculations. These variables (BDE, IP, and PA) do not consider the kinetic factors that can influence the experimental antioxidant capacity, and the steric hindrance in particular can lead to a reduction in the antioxidant capacity [44,45,46]. In this case, the increase in the number of the methyl groups leads to a variation in the electronic density that should favor the antioxidant capacity; however, it also increases the steric hindrance to access the –OH group, which can explain the tendency observed in the ORAC results.

### 2.4. In Vitro and In Silico α-Glucosidase Inhibitory Activity

The effect of synthesized hydroquinones against α-glucosidase activity was evaluated (Table 2). Most of the evaluated compounds showed high inhibitory activity, with six of the ten compounds showing IC_50_ values lower than the well-known AGI acarbose (IC_50_ = 194.0 + 1.7 µM). Compounds **8** and **10** presented the highest inhibitory potency, with IC_50_ values of 60.0 + 1.5 µM and 78.0 + 1.1 µM, respectively, and compound **9** was the worst. These results suggest that the *gem*-diethyl group in ring B is a key structural factor in the inhibitory potency of the compounds, but the presence of methyl substituents in the rest of the structure needs detailed examination due to their variable effect.

**Figure 2:**
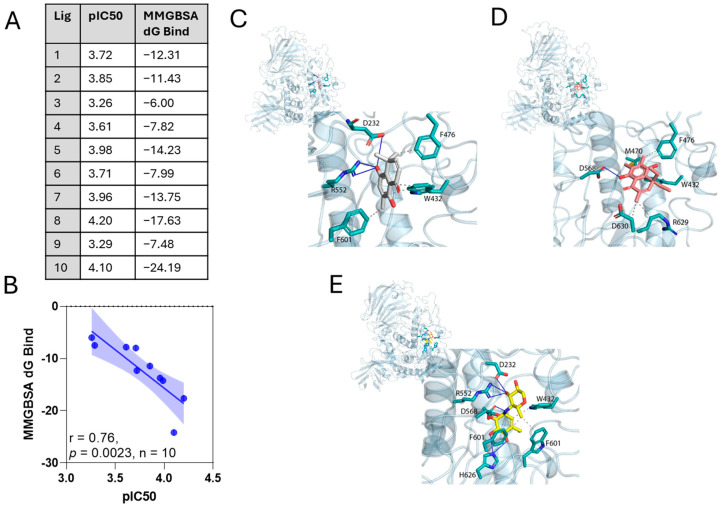
In silico molecular docking for *ortho*-carbonyl hydroquinone series against the α-glucosidase enzyme. (**A**) Binding free energy components for the α-glucosidase–hydroquinone (**3** and **10**) complexes calculated by MM-GBSA analysis; all energies are in kcal/mol and pIC50 for *ortho*-carbonyl hydroquinones. (**B**) Correlations of calculated MMGBSAΔG values with experimental IC_50_ inhibitory values, expressed as pIC50 = −log [IC50]. The r square value from linear regression and *p*-value for Spearman’s correlation (n = 10) are shown. (**C**–**E**) 3D maps binding interactions of compound **3** and **10** against the α-glucosidase enzyme. Ligand compound **3** (**A**,**C**) and compound **10** (**B**,**D**) exposure points are indicated with gray coloring, while the binding interactions of ligands with α-glucosidase are indicated with red lines. (**C**) Binding free energy components for the α-glucosidase–hydroquinone (**3** and **10**) complexes calculated by MM-GBSA analysis; all energies are in kcal/mol.

To obtain a detailed explanation of the structure–activity relationship observed in the experimental inhibitory results, we carried out molecular docking. Several conformers and poses for each molecule were analyzed. The binding site was defined by the crystallographic structure obtained for α-glucosidase in complex with acarbose (PDB code: 3W37). The results were used as an input for an MMGBSA free energy calculation study. Figure 2A shows the values of MMGBSA ΔGbind and the IC_50_ presented as pIC_50_ = –log[IC_50_], and the correlation between them is shown in Figure 2B. The correlation between experimental pIC_50_ values and calculated MMGBSA ΔGbind gives a R2 = 0.71.

Figure 2 shows 3D maps of the binding interactions of compound **3** (Figure 2C), compound **10** (Figure 2D), and acarbose (Figure 2E) with the α-glucosidase enzyme. Acarbose, which corresponds to the model ligand to α-glucosidase, shows a three-center hydrogen bond interaction between one of their hydroxyl groups with aspartic acid 232 and arginine 552, a hydrogen bond interaction between another of their hydroxyls and aspartic acid 568, and a hydrogen bond interaction between two other hydroxyls and histidine 626. Additional hydrophobic interactions of acarbose with tryptophan 432 and phenylalanine 601 are also identified. Compound **3**, which presented the highest MMGBSA ΔG_bind_ values (lowest affinity), presents a three-center hydrogen bond interaction between one of their hydroxyls with aspartic acid 232 and arginine 552, like acarbose, hydrophobic interactions with phenylalanine 601 and phenylalanine 476, and π-π stacking interactions with tryptophan 432. Compound **10**, instead, with the lowest MMGBSA ΔGbind value (highest affinity), shows a hydrogen bond between one of their hydroxyls and aspartic acid 568. In addition, it presents hydrophobic interactions with aspartic acid 630, arginine 629, phenylalanine 476, and methionine 470, and additionally a π-π stacking interaction with tryptophan 432.

MMGBSA binding free energies were estimated for the complex of the enzyme with compounds **3** and **10** (Table 3). Compound **10** shows a stronger net binding energy (ΔG_bind_) compared with compound **3** (−24.19 vs. 6.00 kcal/mol). For both compounds, the Van der Waals energy dominated over all other energies. The favorable contribution of lipophilic and electrostatic binding energy (ΔG_bind_ LP and ΔG_bind_ CB) is similar for both compounds. The most remarkable difference is the unfavorable contribution of the generalized Born electrostatic solvation energy (ΔG_bind_ GB) for compound **3** compared with compound **10**.

### 2.5. Effect of Ortho-Carbonyl Hydroquinones on Proliferation and Metabolism of Caco-2 Cells

**Figure 3:**
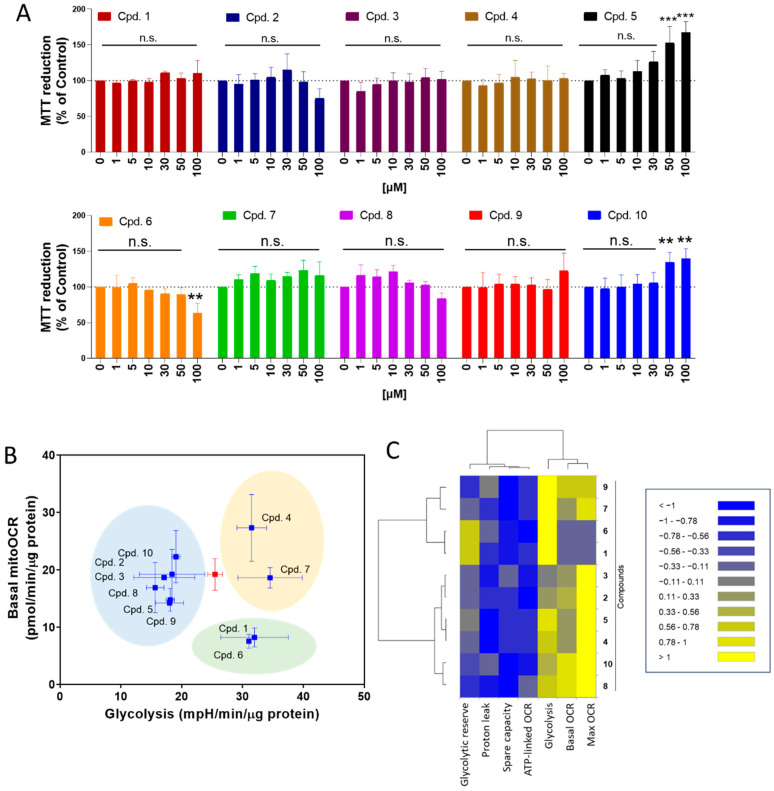
Effect of *ortho*-carbonyl methyl-substituted hydroquinones on proliferation, mitochondrial respiration, and glycolysis in Caco-2 cells. (**A**) Effect of compounds on the MTT reduction in Caco-2. Cells were treated with compounds (1, 5, 10, 30, 50, and 100 µM) for 24 h. (**B**) Dot-plot for basal mitochondrial OCR (basal mitoOCR) vs. glycolysis. The red square represents to control condition. Three groups of compounds are highlighted in blue, yellow, and green. (**C**) Dendrogram with five and two parameters of mitochondrial respiration and glycolysis, respectively, affected by compounds (100 µM) for 24 h of treatment. Data are shown as means ± SD of three independent experiments. ** *p* < 0.01, *** *p* < 0.001 control, and n.s.: not significant.

We evaluate the effect of *ortho*-carbonyl hydroquinones **1**–**10** on the intestinal Caco-2 cell line at 24 h of treatment. As Figure 3A shows, compounds **5** and **10** significantly increased the MTT reduction, suggesting that these compounds increase the cell number. Consistent with the antiproliferative effect previously described by us [30], compound **6**, instead, decreased the MTT reduction. The other compounds of this series did not show significant changes in Caco-2 cell experiments.

We previously described how *ortho*-carbonyl hydroquinone scaffolds inhibit oxidative phosphorylation in breast cancer cells [29,30,31,37]. Since the reduction in glucose utilization and metabolism in intestinal cells is a promising target for T2DM treatment, the effect of these compounds on mitochondrial respiration and glycolysis in Caco-2 cells was determined. As Figure 3B shows, three groups of compounds were identified in a dot-plot from basal mitochondrial OCR and glycolysis (ECAR). Compared to the control (red dot), group A (compounds **2**, **3**, **5**, **8**, **9**, and **10**) reduces the glycolysis. Group B (compounds **1** and **6**) reduces the basal mitoOCR and increases ECAR, suggesting that it induces a metabolic remodeling toward glycolysis, and group C (compounds **4** and **7**) increases the glycolysis lacking effect on OCR. The effect of *ortho*-carbonyl hydroquinone series (100 µM) on five parameters of mitochondrial respiration (basal OCR, max OCR, spare capacity, ATP-linked OCR, and proton leak-driven OCR), and two of glycolysis (glycolytic capacity and glycolysis), is represented in a dendrogram (Figure 3C). Interestingly, two clusters of compounds are shown with differential effects on maximal OCR and glycolysis. Cluster 1 is composed of glycolysis enhancers with a strong inhibitory effect on max. OCR: compounds **1**, **6**, **7**, and **9**. Cluster 2 is composed of glycolysis inhibitors with a reduced effect on max OCR: compounds **2**, **3**, **4**, **5**, **8**, and **10**. No correlations between OCR and ECAR parameters, α-glucosidase activity inhibition, or ORAC were observed (Supplementary Figure S1).

### 2.6. Compounds 5 and 10 Differentially Act on Mitochondrial Respiration, Glycolysis, and ROS Production in Caco-2 Cells

**Figure 4:**
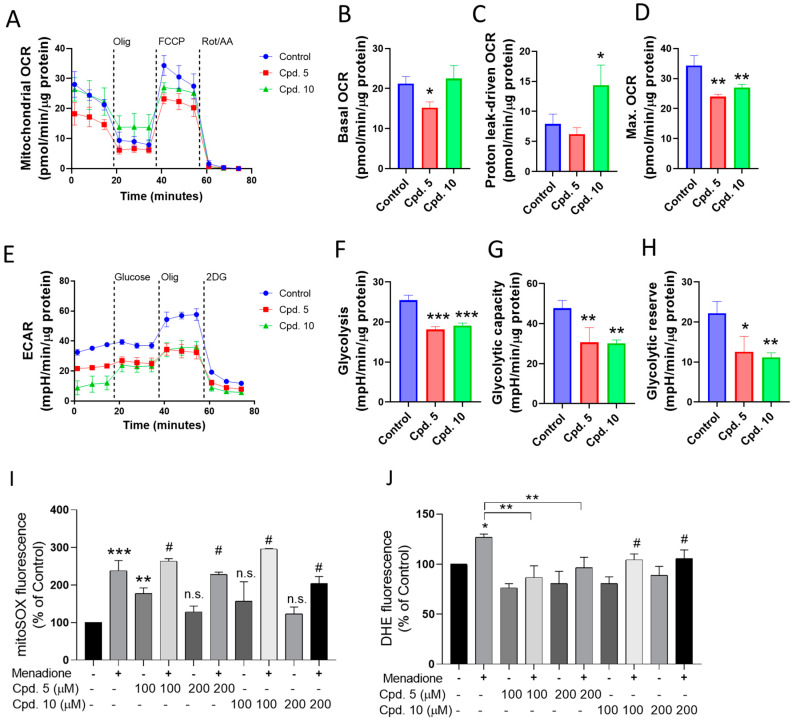
(**A**–**D**) Effect of selected compounds (100 µM, compounds **5** and **10**) on mitochondrial respiration and (**E**–**H**) glycolysis at 24 h of treatment. (**I**,**J**) Evaluation of antioxidant effects in the in vitro model of menadione-dependent ROS production. Effects of compounds **5** and **10** on (**I**) mitochondrial ROS and (**J**) ROS in Caco-2 cells treated with menadione. The mitoROS and cytosolic ROS were evaluated with mitoSOX red and DHE, respectively. Data are shown as means ± SD of three independent experiments. * *p* < 0.05, ** *p* < 0.01, and *** *p* < 0.001, control. n.s.: not significant. #: ** *p* < 0.01 for compound vs. compound + Menadione.

Based on the above results that identify compounds **5** and **10** as among the most active AGIs, we evaluated the effect of both compounds on mitochondrial respiration and glycolysis (Figure 4). Compound **5** reduces the basal and max OCR values without affecting the proton leak-driven OCR. In contrast, compound **10** increases the proton leak-OCR and decreases max OCR (Figure 4A–D). Both compounds significantly reduce the glycolysis (Figure 4E–H).

Using menadione as an ROS inducer [47,48], the preventive effect of compounds **5** and **10** on mitochondrial and cytosolic ROS production was evaluated. No changes in the mitochondrial ROS production were observed (Figure 4I). However, menadione-dependent cytosolic ROS production was significantly prevented by compound **5** (Figure 4J). Collectively, the results suggest that compounds **5** and **10** are AGIs that also reduce glycolysis and mitochondrial respiration, and compound **5** has a cytosolic antioxidant effect.

## 3. Discussion

The T2DM pathogenesis involves alterations in nutrient uptake and energetic metabolism, in which high oxidative stress and inflammation mediate altered cell signaling [49]. Intestinal cells participate in the nutrient uptake and the catabolism of carbohydrates by the α-glucosidase enzyme, which is a current target for T2DM treatment [50]. We evaluated the antioxidant capacity of methyl derivatives of a bicyclic *ortho*-carbonyl hydroquinone, which differ only in the number and position of methyl groups, and their effects as AGIs, and in modulating the energetic metabolism of Caco-2 cells. The antioxidant capacity results suggest that the increase in the methyl groups reduce antioxidant capacity due to the steric hindrance to access the –OH group, despite how the electronic effects should favor it. The docking results suggest that the *gem*-diethyl group in ring B is a key structural factor in the inhibitory potency of the compounds. Compounds **5** and **10** were among the most potent inhibitors of α-glucosidase and produced an increase in the cell number. These compounds correspond to those fully methylated at R_1_, R_2_, and R_3_, with gem dimethyl and gem diethyl at R_4_ and R_5_, respectively (Table 1). Beyond clinical AGI drugs used in T2DM, it has been reported that new naturally occurring compounds act as AGIs [51,52], also producing a reduction in glucose-transporter-encoding gene expression and glucose uptake in vitro and in pre-clinical models [53]. Our results at the cellular level allow us to identify three groups of compounds with differential actions on OXPHOS and the glycolysis of Caco-2 cells. Group A reduced the glycolysis, group B reduced the basal OCR and increased ECAR (metabolic remodeling-inducers), and group C increased the glycolysis lacking effect on OCR. Previously, we described that small structural modifications to the *ortho*-carbonyl hydroquinone scaffold studied produce Complex I inhibitors and OXPHOS uncouplers by a protonophoric mechanism [29,30,31] based on modification in the *gem*-dimethyl/ethyl substitution. Moreover, recent studies on the corresponding quinones showed that these methyl substitutions influence redox potential [54] and the ability to inhibit the mitochondrial electron transport chain [55]. In particular, FRV-1, an *ortho*-carbonyl substituted quinone, was identified as a new inhibitor of the deactive conformational status of Complex I [55]. Notably, our results in Caco-2 cells expand the information available on the structure–mitochondrial activity relationships of *ortho*-carbonyl substituted compounds.

Our results show that in Caco-2 cells, compound **5** was an OCR/ECAR inhibitor and compound **10** was a dual agent, increasing the proton leak-driven OCR and inhibiting the maximal electron transport flux. Despite this different action in OXPHOS, both compounds reduced the glycolysis, suggesting that compounds **5** and **10** could be useful for reducing intestinal glucose uptake and metabolization under a potential T2DM condition.

## 4. Conclusions

We found that the presence of methyl substituents leads to a reduction in the compounds’ antioxidant capacity, and concomitantly an increase in their capacity for AGIs. Fully methylated *ortho*-carbonyl hydroquinones **5** and **10** present lower antioxidant capacity, and potent capacity as AGIs, and they affect the energetic metabolism and reduce glycolysis, promoting the proliferation of Caco-2 cells. Compound **6** decreased the proliferation of Caco-2 cells, while the other compounds in this series did not show significant changes. Compound **5** was an OCR/ECAR inhibitor in Caco-2 cells, while compound **10** was a dual agent, increasing the proton leak-driven OCR and inhibiting the maximal electron transport flux. Our results reveal that slight structural variations in a hydroquinone scaffold led to diverse antioxidant capability, α-glucosidase inhibition, and the regulation of mitochondrial bioenergetics in Caco-2 cells. These findings may be useful for designing new drugs for T2DM and metabolic syndrome.

## 5. Experimental Section

### 5.1. Synthesis and Characterization

#### 5.1.1. General Methods

^1^H and ^13^C NMR spectra were obtained from a spectrometer operating at 400.13 MHz (^1^H) and 100.61 MHz (^13^C) in deuterated chloroform (CDCl_3_) as the solvent. Chemical shifts are reported as ppm downfield from TMS for ^1^H-NMR and relative to the central CDCl_3_ resonance (77.0 ppm) for ^13^C-NMR. All melting points were uncorrected and were determined using an Electrothermal 9100 apparatus. High-resolution mass spectra (HRMS) were obtained on a Bruker Compact Q-TOF MS (ESI/QTOF). Silica gel 60 (230–400 mesh ASTM) and TLC sheets silica gel 60 F254 were used for flash-column chromatography and analytical TLC, respectively. All reagents were purchased from Sigma-Aldrich.

#### 5.1.2. 5,8-Dihydroxy-4,4-dimethylnaphthalen-1(4H)-one (**1**)

This compound was synthesized as previously described [56,57,58,59].

#### 5.1.3. 5,8-Dihydroxy-4,4,6-trimethylnaphthalen-1(4H)-one (**2**) and 5,8-Dihydroxy-4,4,7-trimethylnaphthalen-1(4H)-one (**3**)

Following the general method of acylation, methylhydroquinone and acetic acid react to give a mixture of 3′-Methyl and 4′-methyl 2′,5′-dihydroxyacetophenone regioisomers in a 14:86 ratio and a 32% yield, which was used in the next step without purification. Following the general method, this mixture was oxidized with Ag_2_O and then reacted with 4-(2-methylbut-1-en-1-yl)morpholine to yield compounds **2** and **3**. Column chromatography allows for their separation, in 29% and 5% total yields, respectively. **2** (yellow solid): ^1^H-NMR δ: 1.62 (s, 3H), 2.30 (s, 3H), 4.51 (s, OH), 6.24 (d, *J* = 10.1 Hz, 1H), 6.74 (s, 1H), 6.83 (d, *J* = 10.1 Hz, 1H), 12.72 (s, OH). ^13^C-NMR δ: 16.9, 25.0, 38.2, 113.9, 117.1, 123.9, 132.7, 134.4, 144.1, 156.6, 160.9, 190.9. M.p. 204.0 °C. HRMS (ESI)*m*/*z* calcd. for C_13_H_14_O_3_ [M+H]^+^: 219.1016, found: 219.1019. **3** (yellow solid): ^1^H NMR δ: 1.60 (s, 3H), 2.23 (s, 3H), 4.55 (s, OH), 6.24 (d, *J* = 10.1 Hz, 1H), 6.80 (s, 1H), 6.84 (d, *J* = 10.1 Hz, 1H), 13.01 (s, OH). ^13^C NMR δ: 15.2, 25.0, 38.1, 114.4, 124.1 125.3, 131.7, 144.4, 155.4, 160.9, 191.5. M.p. 230.4 °C (d). HRMS (ESI)*m*/*z* calcd. for C_13_H_14_O_3_ [M+H]^+^: 219.1016, found: 219.1020.

#### 5.1.4. 5,8-Dihydroxy-4,4,6,7-tetramethylnaphthalen-1(4H)-one (**4**) [30,39,60]

^1^H-NMR δ(ppm): 0.54 (s, 3H), 1.58 (dq, *J*_1_ = 14.0 Hz, *J*_2_ = 7.4 Hz, 2H), 2.24 (s, 3H), 2.25 (s, 3H), 2.77 (dq, *J*_1_ = 14.0 Hz, *J*_2_ = 7.5 Hz, 2H), 4.41 (s, OH), 6.46 (d, *J* = 10.13 Hz, 1H), 6.61 (d, *J* = 10.13 Hz, 1H), 13.25 (s, OH).

#### 5.1.5. 5,8-Dihydroxy-2,4,4,6,7-pentamethylnaphthalen-1(4H)-one (**5**)

Column flash chromatography on silica gel allowed for the isolation of **4** (yellow solid, 67% yield). ^1^H-NMR δ(ppm): 1.58 (s, 3H), 1.99 (d, *J* = 1.25 Hz, 3H), 2.23 (s, 3H), 2.25 (s, 3H), 4.41 (s, OH), 6.61 (d, *J* = 1.38 Hz, 1H), 13.39 (s, OH). ^13^C-NMR δ: 11.60, 12.99, 15.72, 25.42, 29.69, 37.34, 112.63, 123.25, 129.76, 131.19, 131.99, 143.35, 155.11, 156.75, 191.55. M.p. 199.9–201.5 °C. HRMS (ESI)*m*/*z* calcd. for C_15_H_18_O_3_ [M+H]^+^: 247.1329, found: 247.1330.

#### 5.1.6. 4,4-Diethyl-5,8-dihydroxynaphthalen-1(4H)-one (**6**)

This compound was synthesized as previously described [30,39,59,60].

#### 5.1.7. 4,4-Diethyl-5,8-dihydroxy-6-methylnaphthalen-1(4H)-one (**7**) and 4,4-Diethyl-5,8-dihydroxy-7-methylnaphthalen-1(4H)-one (**8**)

The 3′- and 4′-methyl acetophenones mentioned above were oxidized with Ag_2_O and then reacted with 4-(2-ethylbut-1-en-1-yl)morpholine to yield compounds *4*,4-diethyl-5,8-dihydroxy-6-methylnaphthalen-1(4H)-one *(**7**)* and 4,4-diethyl-5,8-dihydroxy-7-methylnaphthalen-1(4H)-one (**8**). Column chromatography allows for their separation in 27.5% and 4.5% total yields, respectively. **7** (yellow solid): ^1^H NMR δ: 0.47 (t, *J* = 7.5 Hz, 6H), 1.51 (dq, *J*_1_ = 7.5 Hz, *J*_2_ = 14.0 Hz, 2H), 2.22 (s, 3H), 2.71 (dq, *J*_1_ = 7.5 Hz, *J*_2_ = 14.0 Hz, 3H), 4.40 (s, OH), 6.39 (d, *J* = 10.1 Hz, 1H), 6.55 (d, *J* = 10.1 Hz, 1H) 6.66 (s, 1H), 12.71 (s, OH). ^13^C NMR δ: 9.4, 16.9, 30.6, 48.8, 116.0, 117.2, 128.4, 130.9, 132.2, 143.8, 156.7, 159.4, 191.7. M.p. 198.2 °C. HRMS (ESI)*m*/*z* calcd. for C_15_H_18_O_3_ [M+H]^+^: 247.1329, found: 247.1332. **8** (yellow solid): ^1^H NMR δ: 0.55 (t, *J* = 7.5 Hz, 6H), 1.57 (dq, *J*_1_ = 7.5 Hz, *J*_2_ = 14 Hz, 2H), 2.23 (s, 3H), 2.75 (dq, *J*_1_ = 7.5 Hz, *J*_2_ = 14 Hz, 2H), 4.50 (s, OH), 6.48 (d, *J* = 10,1 Hz, 1H), 6.64 (d, *J* = 10.1 Hz, 1H), 6.77 (s, 1H), 13.08 (s, OH). ^13^C NMR δ: 9.4, 15.2, 30.6, 48.5, 117.0, 124.8, 125.2, 128.1, 128.3, 144.2, 155.8, 159.6, 190.9. M.p. 125.3–129.0 °C. HRMS (ESI)*m*/*z* calcd. for C_15_H_18_O_3_ [M+H]^+^: 247.1329, found: 247.1330.

#### 5.1.8. Synthesis of 4,4-Diethyl-5,8-dihydroxy-6,7-dimethylnaphthalen-1(4H)-one (9)

Column flash chromatography on silica gel allowed for the isolation of **8** (yellow solid, 62% yield). ^1^H NMR δ: 0.54 (t, *J* = 7.5 Hz, 6H), 1.57 (dq, *J*_1_ = 7.5 Hz, *J*_2_ = 14.0 Hz, 2H), 2.24 (s, 3H), 2.25 (s, 3H), 2.77 (dq, *J*_1_ = 7.5 Hz, *J*_2_ = 14.0 Hz, 2H), 4.40 (s, OH), 6.46 (d, *J* = 10.1 Hz, 1H), 6.61 (d, *J* = 10.1 Hz, 1H), 13.25 (s, OH). ^13^C NMR δ: 9.44, 11.60, 13.06, 30.77, 48.57, 115.32, 123.52, 127.95, 128.53, 131.17, 143.30, 155.32, 159.21, 191.83. M.p. 174.9–179.8 °C. HRMS (ESI)*m*/*z* calcd. for C_16_H_20_O_3_ [M+H]^+^: 261.1485, found: 261.1489.

#### 5.1.9. Synthesis of 4,4-Diethyl-5,8-dihydroxy-2,6,7-trimethylnaphthalen-1(4H)-one (**10**)

Column flash chromatography on silica gel allowed for the isolation of **8** (yellow solid, 67% yield). ^1^H NMR δ: 0.51 (t, *J* = 7.44, 3H), 1.55 (dq, *J*_1_ = 7.5 Hz, *J*_2_ = 13.5 Hz, 2H), 2.04 (d, *J* = 1.1 Hz, 3H), 2.23 (s, 3H), 2.24 (s, 3H), 2.72 (dq, *J*_1_ = 7.5 Hz, *J*_2_ = 13.5 Hz, 2H), 4.35 (s, OH), 6.41 (d, *J* = 1.1, 1H), 13.45 (s, OH). ^13^C NMR δ: 9.48, 11.64, 13.01, 15.69, 31.00, 47.76, 115.28, 123.29, 128.36, 130.69, 134.36, 143.14, 155.13, 155.24, 192.26. M.p. 186.1–187.2 °C. HRMS (ESI)*m*/*z* calcd. for C_17_H_22_O_3_ [M+H]^+^: 275.1642, found: 275.1643.

### 5.2. Antioxidant and Enzymatic Inhibitory Assays

#### 5.2.1. ORAC Assays

The antioxidant activity was determined by ORAC (oxygen radical absorbance capacity) assays [61]. Briefly, the antioxidant capacity for ORAC-FL was estimated by measuring the changes in fluorescence after 120 min of reaction with the radical. Assays were performed using a 96-well microplate in a Synergy H1 hybrid multi-mode microplate reader (Biotek, Winooski, VT, USA), and the results were expressed as µmol Trolox 100 g^−1^ DW. Three replicates were accomplished for each analysis.

#### 5.2.2. α-Glucosidase Inhibitory Activity

The inhibition of α-glucosidase was determined following a previous method [62] using *p*-nitrophenyl α-D-glucopyranoside as a substrate. The reaction mixture contained 160 µL of 0.1 M sodium phosphate buffer (pH = 6.9), 5 µL of enzyme (5.46 U/mL), and polyphenolic extract (0.17–1.36 µg/mL). After the pre-incubation of the reaction mixture on ice for 5 min, the enzyme reaction was started by adding 5 µL of 25 mM *p*-nitrophenyl α-D-glucopyranoside into this mixture. The reaction was incubated for 15 min at 37 °C. Then, 80 µL of 0.2 M sodium carbonate was added. The absorbance was measured at 405 nm in a microplate reader (Biotek ELx808, Agilent, Santa Clara, CA, USA). Enzyme inhibition was calculated using the following equation: % inhibition = (A_0_ − A_s_)/A_0_ × 100, where A_0_ is the absorbance of the control (blank, without extract) and A_s_ is the absorbance in presence of the extract. IC_50_ values denote the µg GAE/mL required to inhibit the enzyme by 50%.

### 5.3. Quantum Chemical Calculations

Calculations were achieved using the Gaussian 09 software [63], at density functional theory (DFT) M06-2X/6-311+G(d,p) level. No imaginary vibrational frequencies were found at the optimized geometries, indicating that they are the true minimal of the potential energy surface. The calculated thermodynamic parameters were obtained as follows:

- Bond dissociation enthalpy (BDE): H(RO^•^) + H(H^•^) − H(ROH);
- Ionization potential (IP): H(ROH^•+^) + H(e^−^) − H(ROH);
- Proton affinity (PA): H(RO^−^) + H(H^+^) − H(ROH);
- Electron transfer enthalpy (ETE): H(RO^•^) + H(e^−^) − H(RO^−^).

where H(RO^•^), H(ROH), and H(ROH^•+^) correspond to the enthalpies of the neutral radical and the neutral and radical cation H(RO^−^) of the compounds, while H(e^−^), H(H^+^) and H(H^•^) correspond to enthalpies of the electron, proton, and radical hydrogen.

### 5.4. Molecular Docking

Molecules **1** to **10** were sketched and prepared using Maestro and LigPrep (Schrödinger Release 2022-1: Maestro, LigPrep Schrödinger, LLC, New York, NY, USA, 2021), generating all molecules without adding charges. ConfGen [64] was used to generate at most 10 conformers per molecule. Protein–ligand docking was performed using Glide software (version 9.0, Schrödinger, LLC, New York, NY, USA, 2021) [65,66]. The binding site was defined by the crystallographic structure obtained for alpha-glucosidase in a complex with acarbose (PDB code: 3W37). Before docking calculations, the protein was prepared using Maestro Protein Preparation Wizard [67] by removing ligands, metals, and water molecules; adding hydrogens; and ionizing residues at pH 7.4. the minimization of the protein structure was conducted with an OPLS4 force field. The molecules were then prepared using the OPLS force field to minimize energy. The grid box was defined using the ligand cocrystallized in α-glucosidase as the center of the box. The docking was performed with the Glide standard precision (SP) function [68] and the top-10 poses per docked ligands were selected and subjected to postprocessing and rescoring with the molecular mechanics-generalized Born surface area (MM-GBSA) with Prime [69]. This computational method combines molecular mechanics energy and implicit solvation models, which enable the docking results to be rescored and correlated with the experimental activities (IC_50_). The predicted binding energies (ΔG_bind_) between the ligands and the receptor were calculated as previously reported [70].

### 5.5. Cell Culture Conditions

Caco-2 cells were grown in Dulbecco’s modified Eagle’s Medium/Nutrient Mixture F-12 (D-MEM/F-12), supplemented with 5% fetal bovine serum (FBS), penicillin (100 IU/mL), streptomycin (100 μg/mL), and nonessential amino acid solution 1% (GIBCO, Thermo Fisher Scientific, Grand Island). The culture media contained no exogenous pyruvate supplementation, and the cells were maintained in a humidified atmosphere at 37 °C and 5% CO_2_.

### 5.6. MTT Assay

The cell proliferation was determined using the MTT assay. The cells were incubated in 96-well plates at 7000 cells per well and incubated for 24 h. The cells were treated for 24 h with compounds (1, 5, 10, 30, 50, and 100 μM). After treatment, the culture medium was removed, and the cells were incubated with MTT for 1 h at 37 °C. Finally, 100 μL of DMSO was added and measured by spectrophotometry at 570 nm, as previously described [71].

### 5.7. Determination of Extracellular Acidification Rate and Oxygen Consumption Rate in Real-Time

To analyze the extracellular acidification rate (ECAR), Caco-2 cells (10,000 cells/well) were seeded on XFe96 V3-PS multi-well plates and kept for 24 h at 37 °C in 5% CO_2_ with a DMEM culture medium supplemented with FBS. After 24 h, the cells were stimulated by compounds at 100 μM for 24 h. Then, the culture medium was replaced with assay media (unbuffered DMEM without red phenol, with 4 mM glutamine and 10 mM glucose, pH = 7.4) 1 h before the assay. Glycolysis was evaluated by the sequential injection of 10 mM glucose, 1 µM oligomycin, and 100 mM 2-deoxy-D-glucose (2-DG), and ECAR was analyzed using specific excitation and emission wavelengths of protons (470/530 nm). To study the mitochondrial function, the sequential injection of 1 μM oligomycin, 250 nM FCCP, 1 μM rotenone, and 1 μM antimycin A was added. The oxygen consumption rate (OCR) measurements were made with the specific excitation and emission wavelengths of the fluorescent probes for oxygen (532/650 nm) using a Seahorse XFe96 Analyzer (Seahorse Agilent, USA), and OCR and ECAR data were normalized by protein content/well, which was determined using a BCA kit [55]. Each experiment was performed at least in triplicate. The OCR/ECAR data analysis and dendrograms were performed using XLSTAT software (version 2023.2.1414, Lumivero, New York, NY, USA).

### 5.8. Determination of Cytosolic and Mitochondrial ROS (mtROS)

The mtROS and ROS levels were measured using MitoSOX^®^ Red Mitochondrial Superoxide (Invitrogen, Carlsbad, CA, USA) and Invitrogen™ Dihydroethidium (Hydroethidine, DHE) probes, respectively. The Caco-2 cells were incubated in 12-well plates at 50,000 cells for 24 h. The cells were treated with compounds **5** and **10** at 100 μM and 200 μM for 24 h. Next, the cells were incubated with 25 μM menadione 25 μM for 1 h, and then the cells were washed with PBS and incubated with MitoSOX Red^®^ (5 µM) or DHE (5 μM) for 30 min. Then, they were recollected, and washed, and the fluorescence was detected by flow cytometry according to Urra et al., 2018 [31].

### 5.9. Statistics

The data are expressed as the mean ± standard deviation (SD) of three independent experiments. Statistical analysis was performed using one-way or two-way ANOVA with Bonferroni’s post-test for pairwise comparisons with Graph Pad Prism 4.03 (GraphPad Software, San Diego, CA, USA). To study the differences between variables, the Pearson correlation coefficient was used. The data were considered statistically significant when *p* < 0.05.

## Figures and Tables

**Table 1:** Compounds studied in this work.

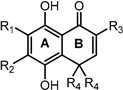				
Compound	R1	R2	R3	R4
**1**	-H	-H	-H	-CH_3_
**2**	-H	-CH_3_	-H	-CH_3_
**3**	-CH_3_	-H	-H	-CH_3_
**4**	-CH_3_	-CH_3_	-H	-CH_3_
**5**	-CH_3_	-CH_3_	-CH_3_	-CH_3_
**6**	-H	-H	-H	-CH_2_CH_3_
**7**	-H	-CH_3_	-H	-CH_2_CH_3_
**8**	-CH_3_	-H	-H	-CH_2_CH_3_
**9**	-CH_3_	-CH_3_	-H	-CH_2_CH_3_
**10**	-CH_3_	-CH_3_	-CH_3_	-CH_2_CH_3_

**Table 3:** Calculated binding energies of molecules **3** and **10** in the complex.

Compound	MM-GBSA (kcal/mol)								
	Δ*G*_bind_	Δ*G*_bind CB_	Δ*G*_bind CV_	Δ*G*_bind HB_	Δ*G*_bind LP_	Δ*G*_bind PK_	Δ*G*_bind SC_	Δ*G*_bind GB_	Δ*G*_bind VW_
**3**	−6	−6.55	0.07	−1.17	−12.19	−1.86	0.02	38.33	−22.65
**10**	−24.19	−8.02	6.67	−1	−16.26	−0.82	0	18.33	−23.09

## Data Availability

Data is contained within the article or Supplementary Material.

## Supplementary Materials

The following supporting information can be downloaded at https://www.mdpi.com/article/10.3390/ijms25158334/s1.
