# Supplementary material for: Identification of Antioxidant Methyl Derivatives of Ortho-Carbonyl Hydroquinones That Reduce Caco-2 Cell Energetic Metabolism and Alpha-Glucosidase Activity
Source: Int J Mol Sci. 2024 Jul 30;25(15):8334. doi: 10.3390/ijms25158334 (PMC11313435; doi:10.3390/ijms25158334)
Supplement: Supplementary file 1 [file ijms-25-08334-s001.zip › ijms-3121641-supplementary.pdf]

Identification of Antioxidant Methyl Derivatives of *Ortho*-Carbonyl Hydroquinones That Reduce Caco-2 Cell Energetic Metabolism and Alpha-Glucosidase Activity

Matías Monroy-Cárdenas, Cristopher Almarza, Paulina Valenzuela-Hormazábal, David Ramírez, Félix A. Urra, Maximiliano Martínez-Cifuentes * and Ramiro Araya-Maturana *

SUPPLEMENTARY INFORMATION

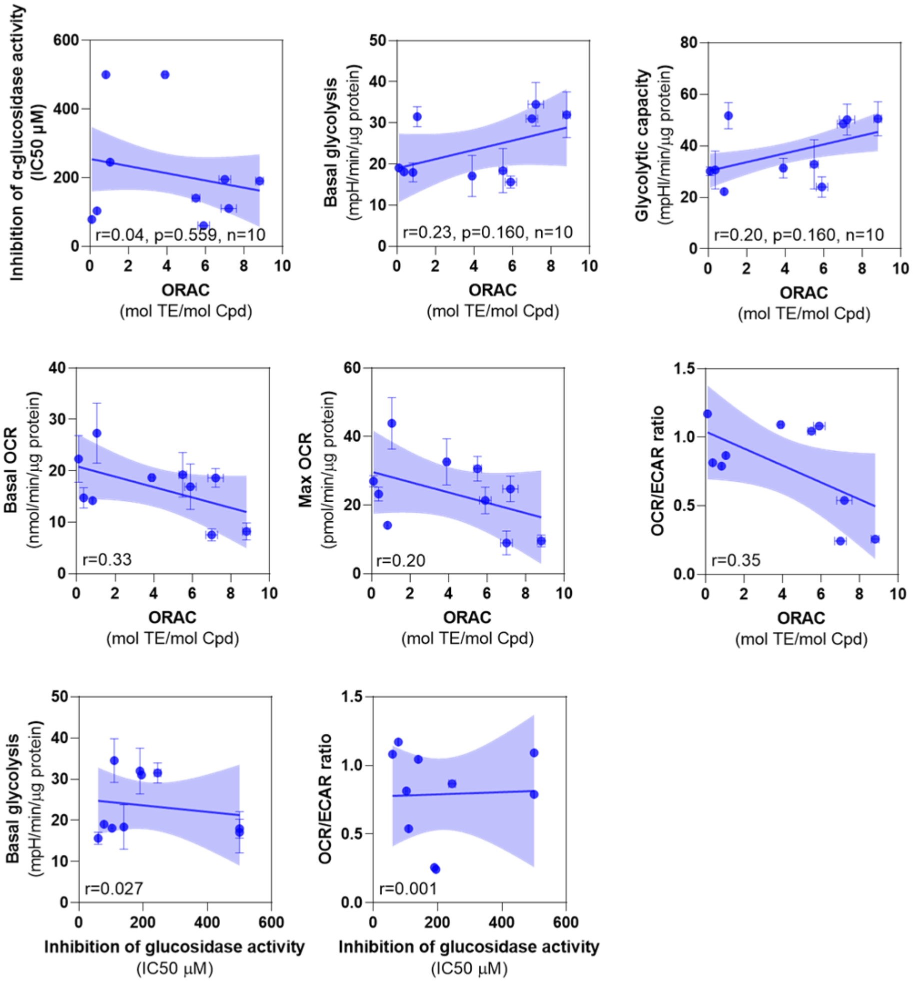

Supplementary Figure S1. Relationships between mitochondrial respiration and glycolysis parameters, inhibition of α-glucosidase activity inhibition and ORAC. The r-square value from linear regression and the p-value for Spearman's correlation (n = 10) are shown.

Data Spectra

5,8-dihydroxy-4,4,6-trimethylnaphthalen-1(4H)-one **(Compound 2)**

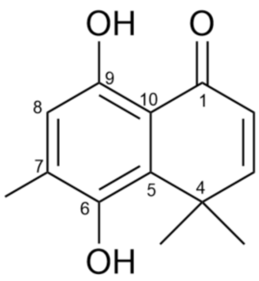

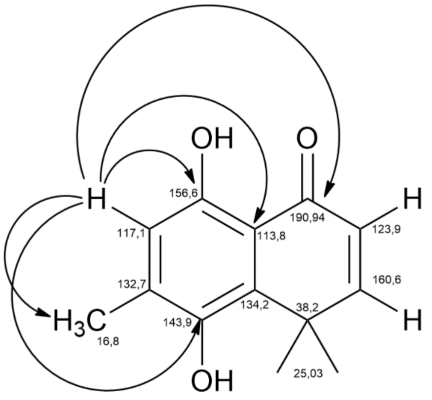

	desplazamiento 1H	desplazamiento 13C	acoplamiento
1	-	190,9	-
2	6,24	123,9	C-4; C-10
3	6,83	160,6	Me-4; Me'-4; C-4; C-5; C-1
4	-	38,2	-
Me-4	1,62	24,9	C-4; C-5; C-3
5	-	134,2	-
6	-	143,9	-
7	-	132,8	-
8	6,74	117,1	Me-7; C-7; C-9; C-10, C-1
9	-	156,6	-
10	-	113,8	-

1H

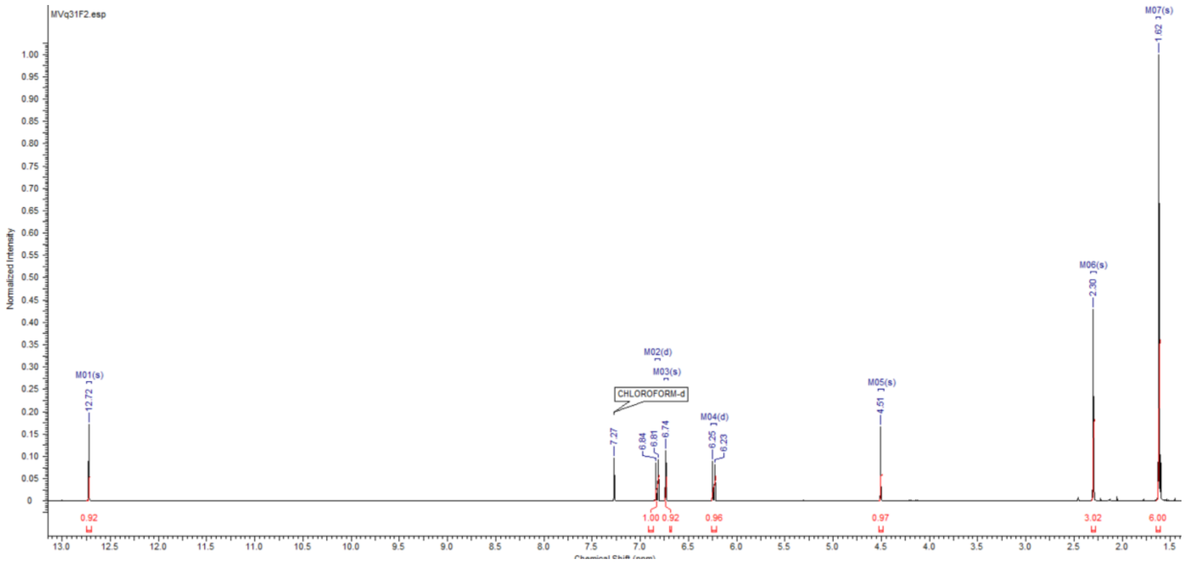
MVq31F2.esp
Normalized Intensity
M01(s)
12.72
CHLOROFORM-d
7.27
M02(d)
6.84
6.81
M03(s)
6.74
M04(d)
6.25
6.23
M05(s)
4.51
M06(s)
2.30
M07(s)
1.62
0.92
1.00
0.92
0.96
0.97
3.02
6.00

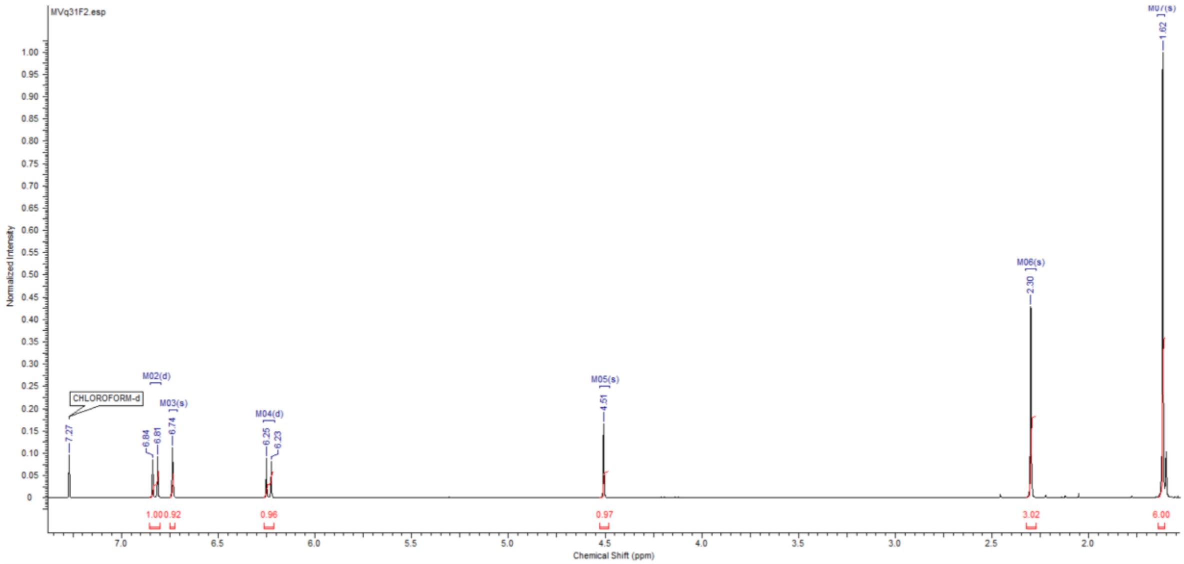
MVq31F2.esp
Normalized Intensity
CHLOROFORM-d
7.27
M02(d)
6.84
6.81
M03(s)
6.74
M04(d)
6.25
6.23
M05(s)
4.51
M06(s)
2.30
M07(s)
1.62
1.00
0.92
0.96
0.97
3.02
6.00
Chemical Shift (ppm)

13C

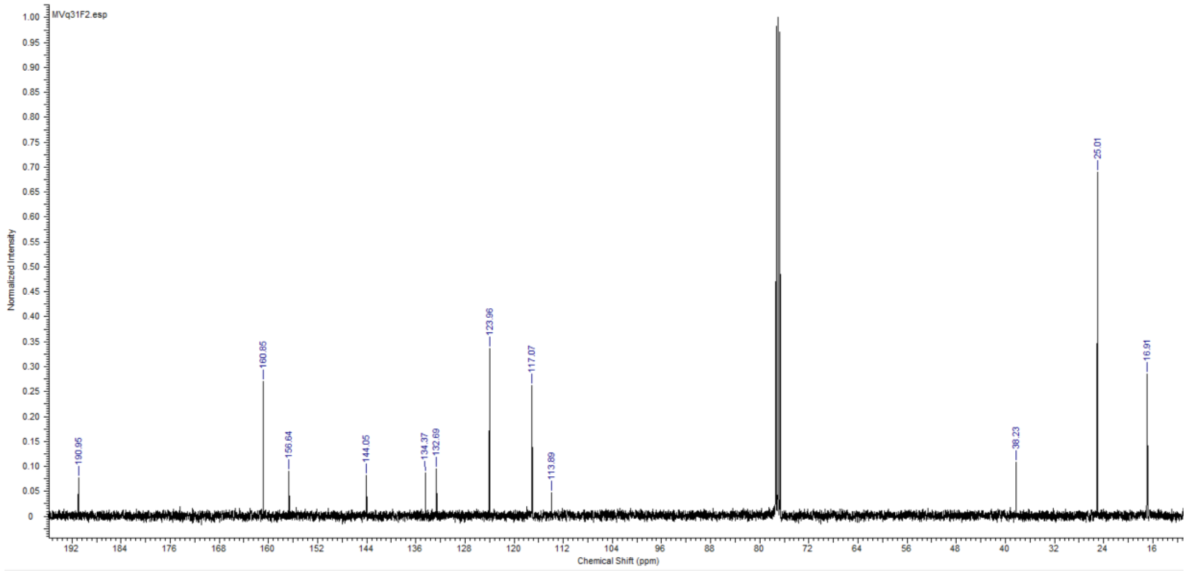
MVq31F2.esp
Normalized Intensity
Chemical Shift (ppm)
190.95
160.85
156.64
144.05
134.37
132.69
123.96
117.07
113.89
38.23
25.01
16.91

HSQC

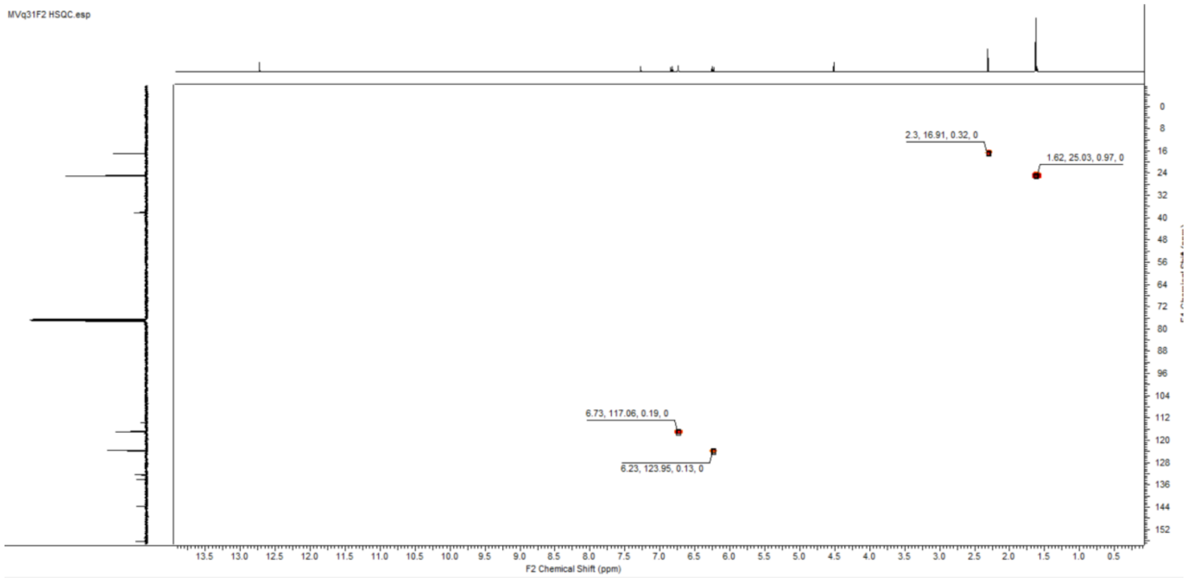
MVq31F2 HSQC.esp
2.3, 16.91, 0.32, 0
1.62, 25.03, 0.97, 0
6.73, 117.06, 0.19, 0
6.23, 123.95, 0.13, 0
F2 Chemical Shift (ppm)

HMBC

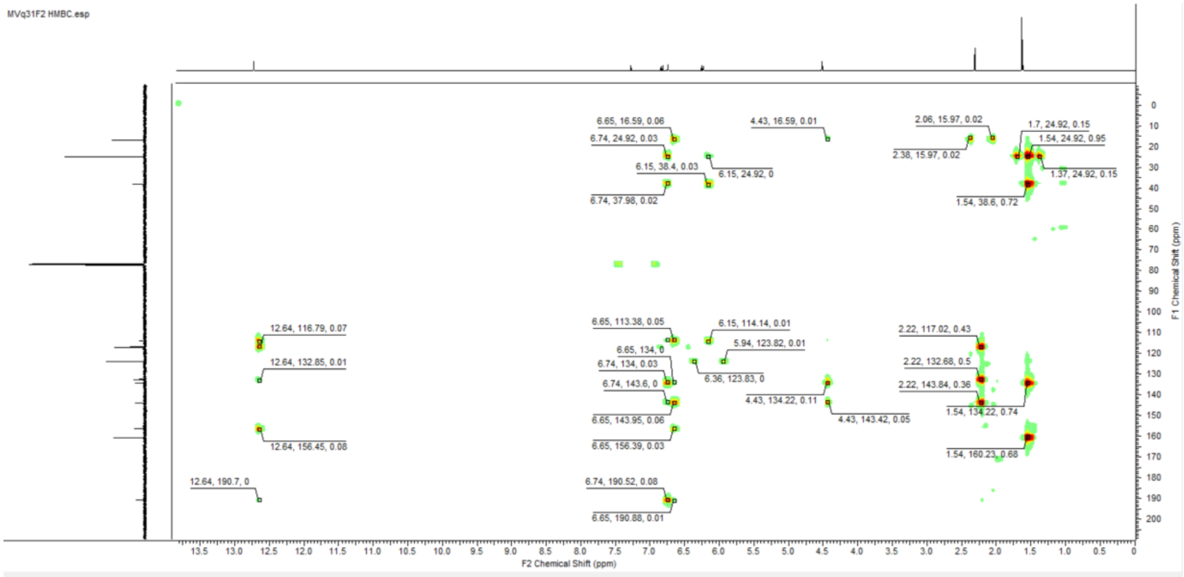

MVq31F2 HMBC.esp
6.65, 16.59, 0.06
6.74, 24.92, 0.03
6.15, 38.4, 0.03
6.15, 24.92, 0
6.74, 37.98, 0.02
4.43, 16.59, 0.01
2.06, 15.97, 0.02
2.38, 15.97, 0.02
1.7, 24.92, 0.15
1.54, 24.92, 0.95
1.37, 24.92, 0.15
1.54, 38.6, 0.72
12.64, 116.79, 0.07
12.64, 132.85, 0.01
12.64, 156.45, 0.08
12.64, 190.7, 0
6.65, 113.38, 0.05
6.65, 134, 0
6.74, 134, 0.03
6.74, 143.6, 0
6.65, 143.95, 0.06
6.65, 156.39, 0.03
6.74, 190.52, 0.08
6.65, 190.88, 0.01
6.15, 114.14, 0.01
5.94, 123.82, 0.01
6.36, 123.83, 0
4.43, 134.22, 0.11
4.43, 143.42, 0.05
2.22, 117.02, 0.43
2.22, 132.68, 0.5
2.22, 143.84, 0.36
1.54, 134.22, 0.74
1.54, 160.23, 0.68
F2 Chemical Shift (ppm)
F1 Chemical Shift (ppm)

HRMS

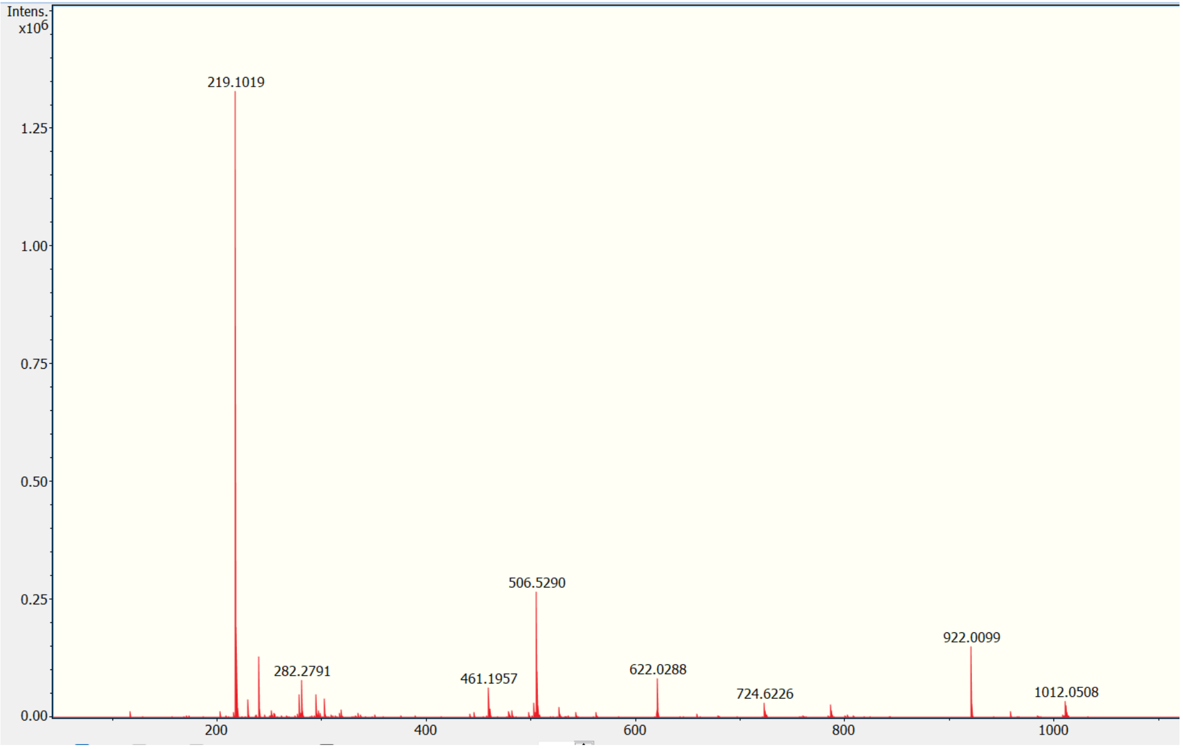

Intens.
x10^6
219.1019
282.2791
461.1957
506.5290
622.0288
724.6226
922.0099
1012.0508

5,8-dihydroxy-4,4,7-trimethylnaphthalen-1(4H)-one **(Compound 3)**

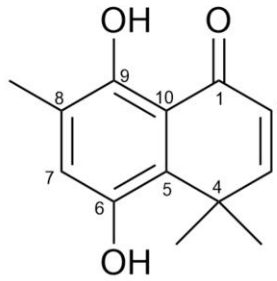

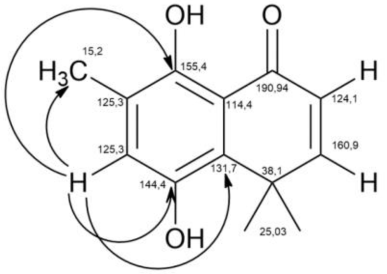

	δ ^{1}H	δ ^{13}C	acoplamiento
1	-	191,5	-
2	6,25	124,1	C-4; C-10
3	6,84	160,9	Me-4; Me'-4; C-4; C-5; C-1
4	-	38,1	-
Me-4	1,60	25	C-4; C-5; C-3
5	-	131,7	-
6	-	144,4	-
7	6,80	125,3	Me-8; C-5; C-6; C-9
8	-	125,3	-
9	-	155,4	-
10	-	114,4	-
Me-8	2,23	15,2	C-7 o C-8; C-9
OH-6	4,55	-	C-5; C-6
OH-9	13,01	-	C-10; C8; C-9

1H

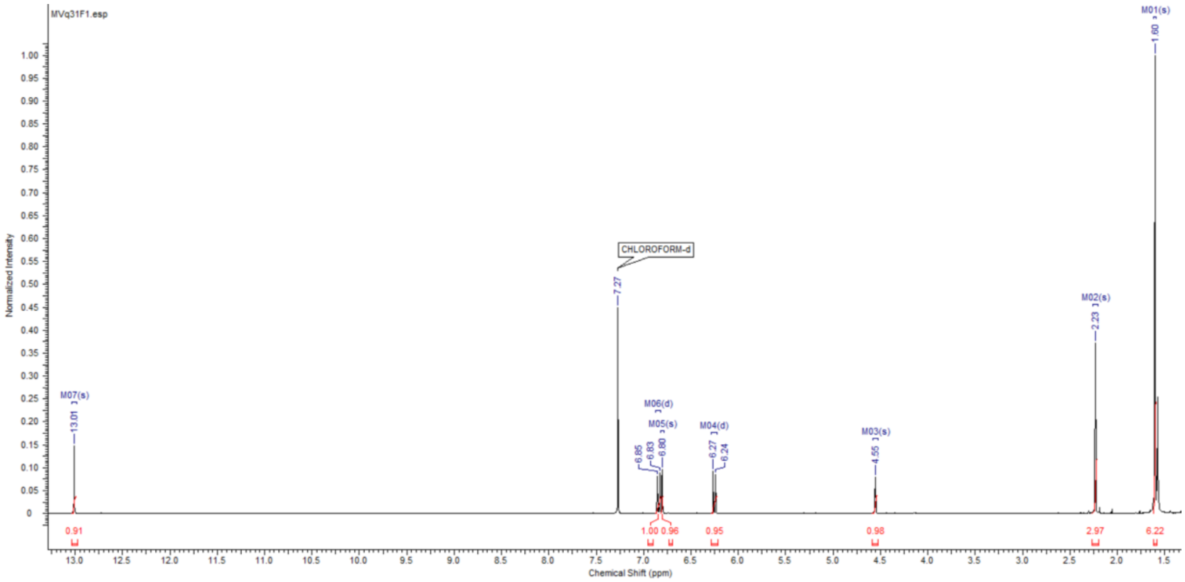
MVq31F1.esp
CHLOROFORM-d
M01(s)
M02(s)
M03(s)
M04(d)
M05(s)
M06(d)
M07(s)
Normalized Intensity
Chemical Shift (ppm)

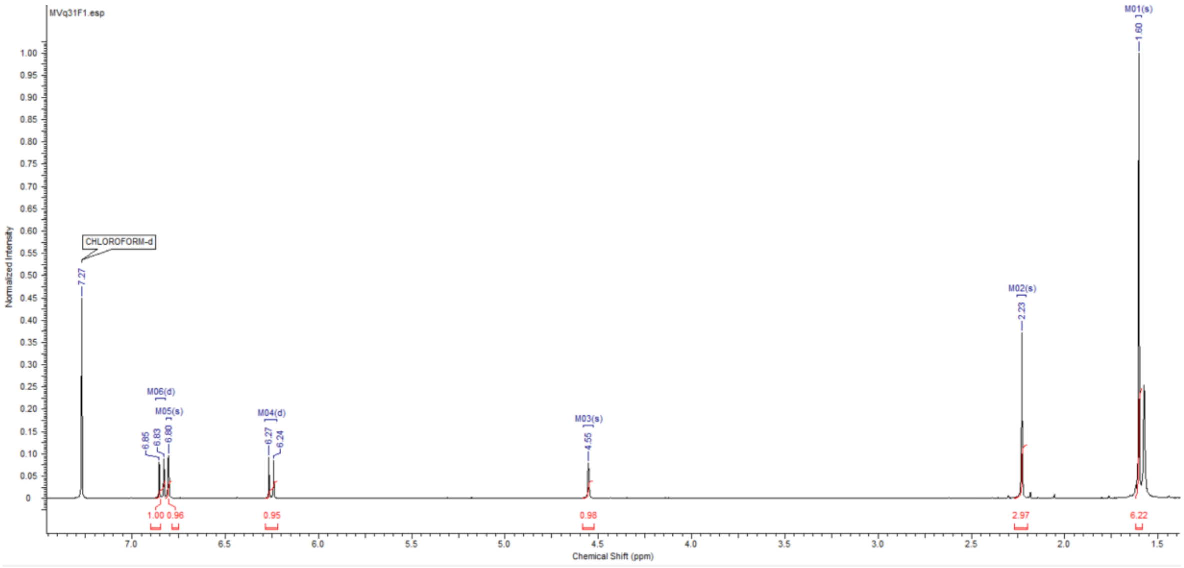
MVq31F1.esp
CHLOROFORM-d
M01(s)
M02(s)
M03(s)
M04(d)
M05(s)
M06(d)
Normalized Intensity
Chemical Shift (ppm)

13C

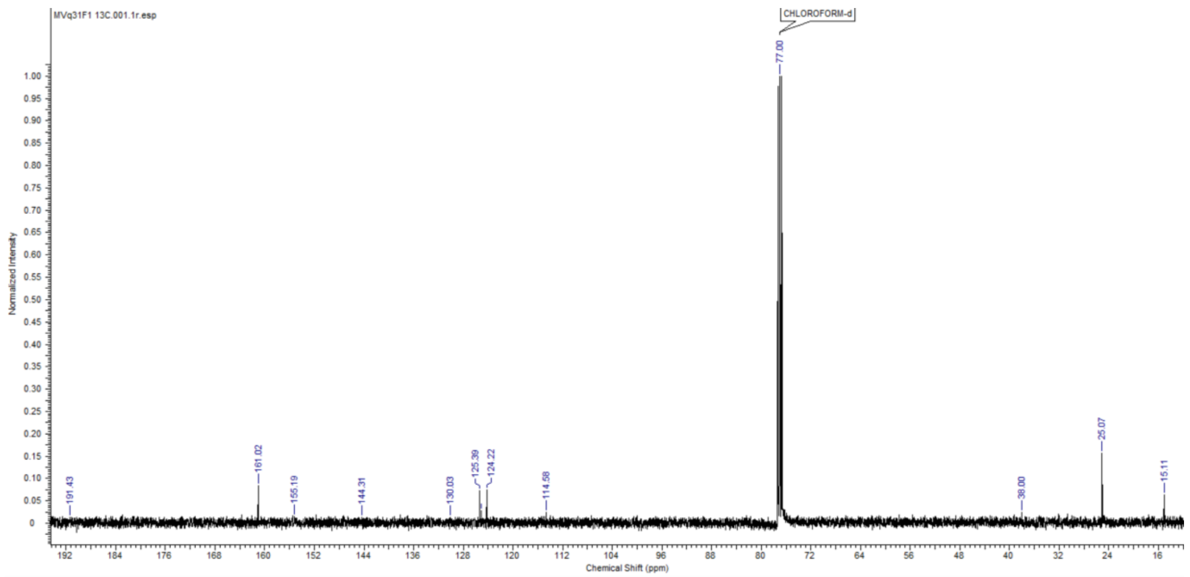
MVq31F1 13C.001.1r.esp
CHLOROFORM-d
77.00
191.43
161.02
155.19
144.31
130.03
125.39
124.22
114.58
38.00
25.07
15.11
Normalized Intensity
Chemical Shift (ppm)

HSQC

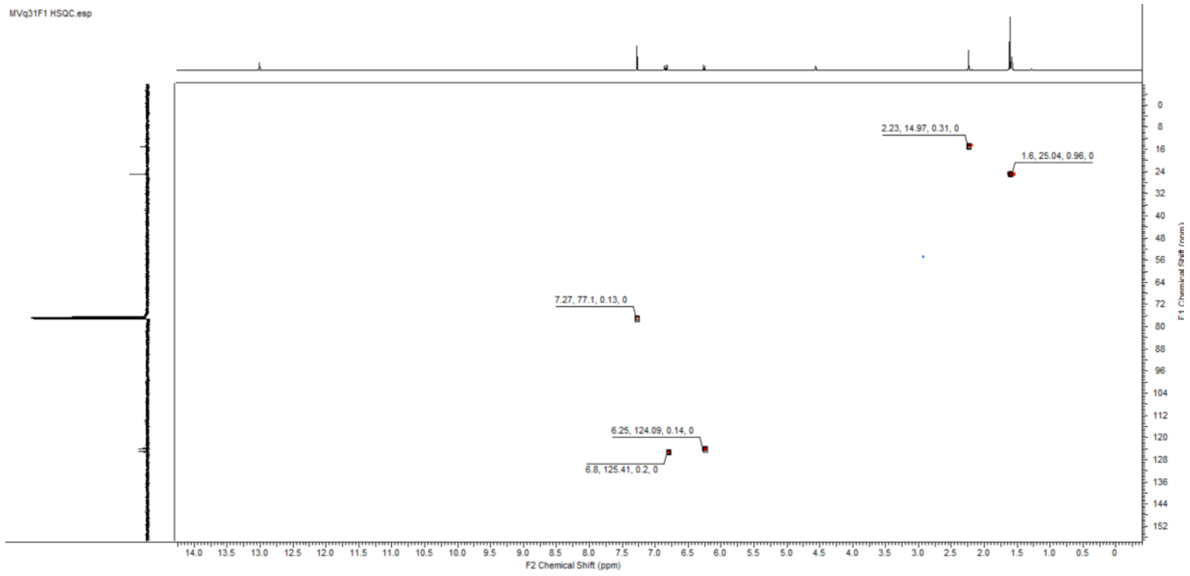
MVq31F1 HSQC.esp
2.23, 14.97, 0.31, 0
1.6, 25.04, 0.96, 0
7.27, 77.1, 0.13, 0
6.25, 124.09, 0.14, 0
6.8, 125.41, 0.2, 0
F2 Chemical Shift (ppm)
F1 Chemical Shift (ppm)

HMBC

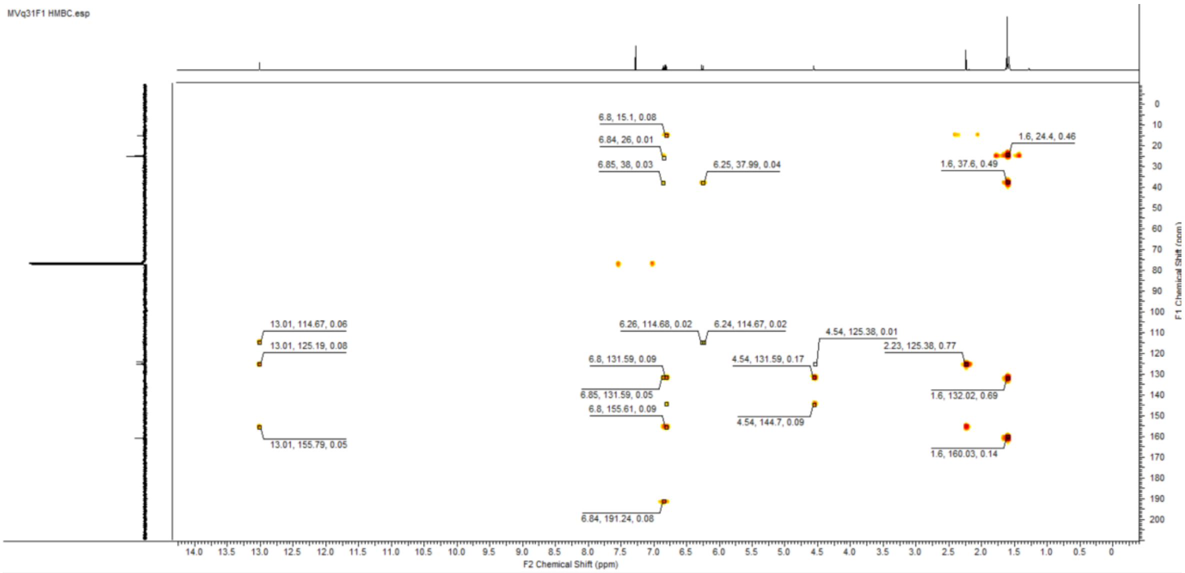
MVq31F1 HMBC.esp
6.8, 15.1, 0.08
6.84, 26, 0.01
6.85, 38, 0.03
6.25, 37.99, 0.04
1.6, 24.4, 0.46
1.6, 37.6, 0.49
13.01, 114.67, 0.06
13.01, 125.19, 0.08
6.26, 114.68, 0.02
6.24, 114.67, 0.02
4.54, 125.38, 0.01
2.23, 125.38, 0.77
6.8, 131.59, 0.09
4.54, 131.59, 0.17
6.85, 131.59, 0.05
6.8, 155.61, 0.09
1.6, 132.02, 0.69
4.54, 144.7, 0.09
13.01, 155.79, 0.05
1.6, 160.03, 0.14
6.84, 191.24, 0.08
F2 Chemical Shift (ppm)
F1 Chemical Shift (ppm)

HRMS

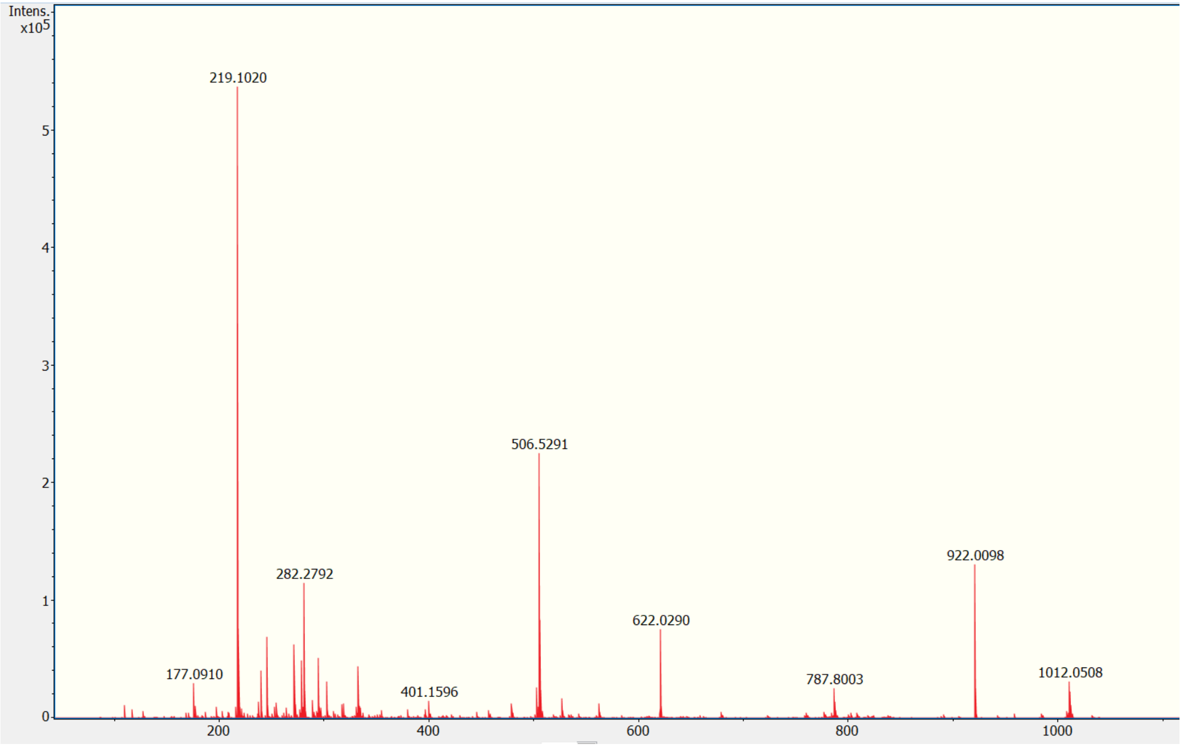
Intens.
x10^5
219.1020
506.5291
282.2792
922.0098
622.0290
177.0910
401.1596
787.8003
1012.0508

5,8-dihydroxy-2,4,4,6,7-pentamethylnaphthalen-1(4H)-one **(compound 5)**

1H

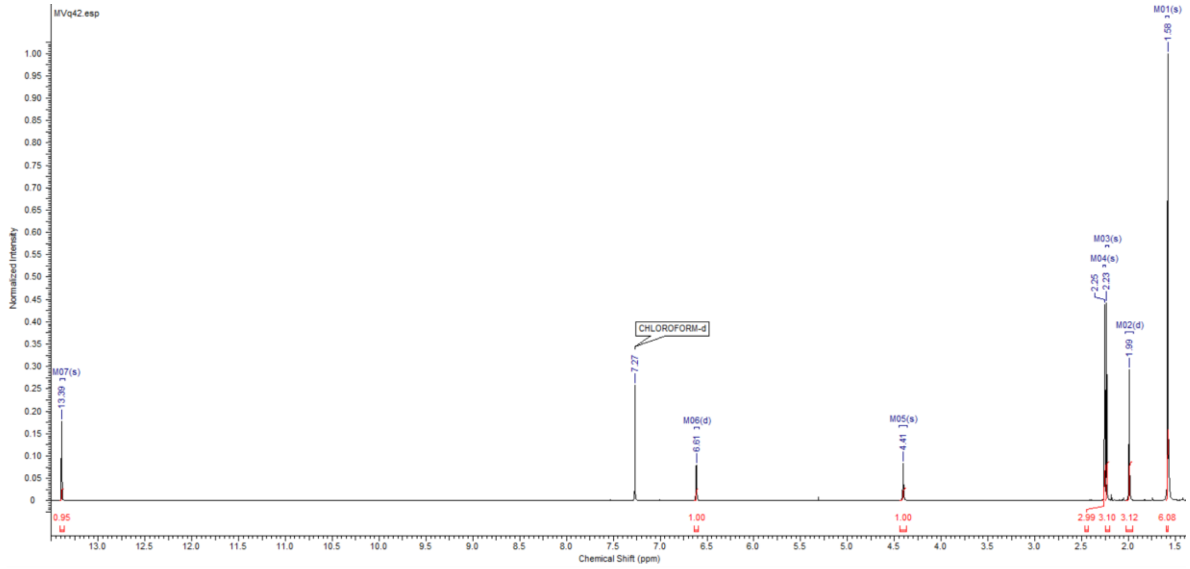

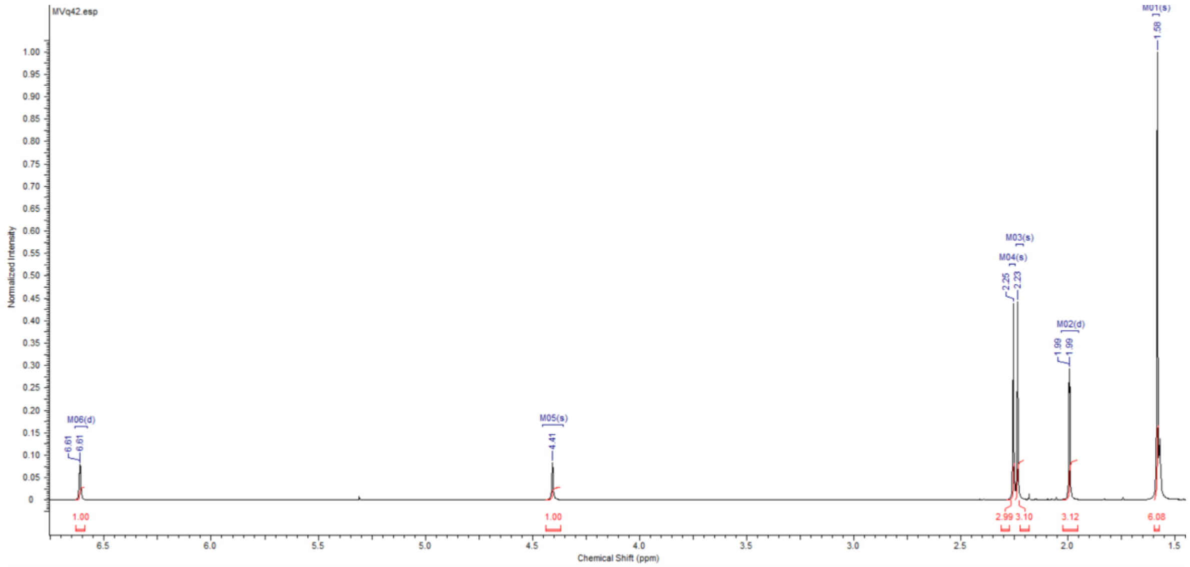

13C

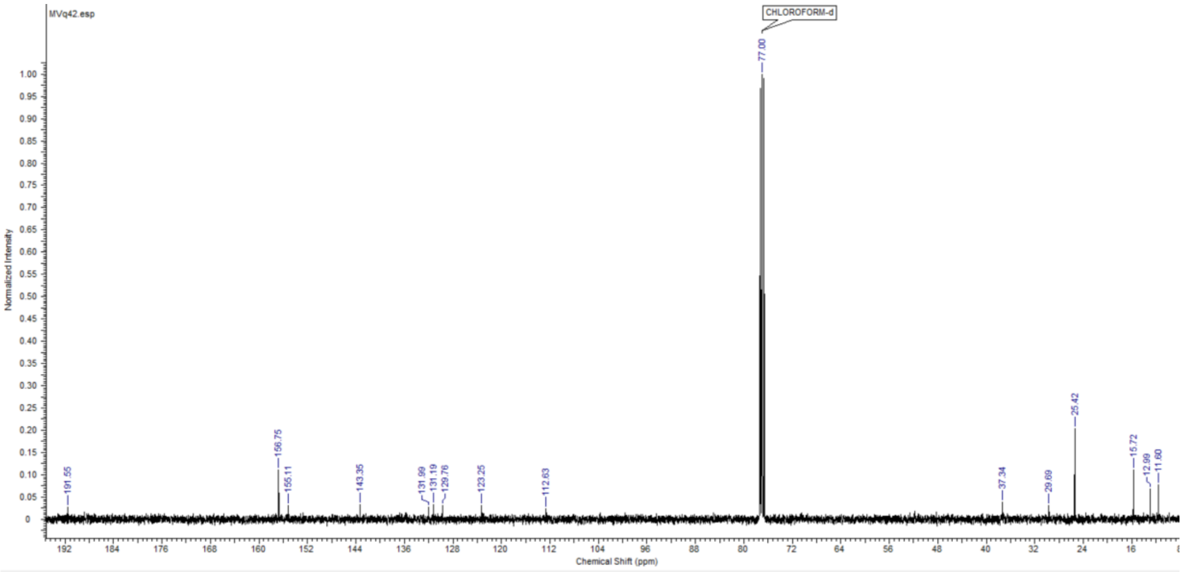
MVq42.esp
CHLOROFORM-d
191.55
156.75
155.11
143.35
131.99
131.19
129.76
123.25
112.63
77.00
37.34
29.69
25.42
15.72
12.99
11.60
Normalized Intensity
Chemical Shift (ppm)

HRMS

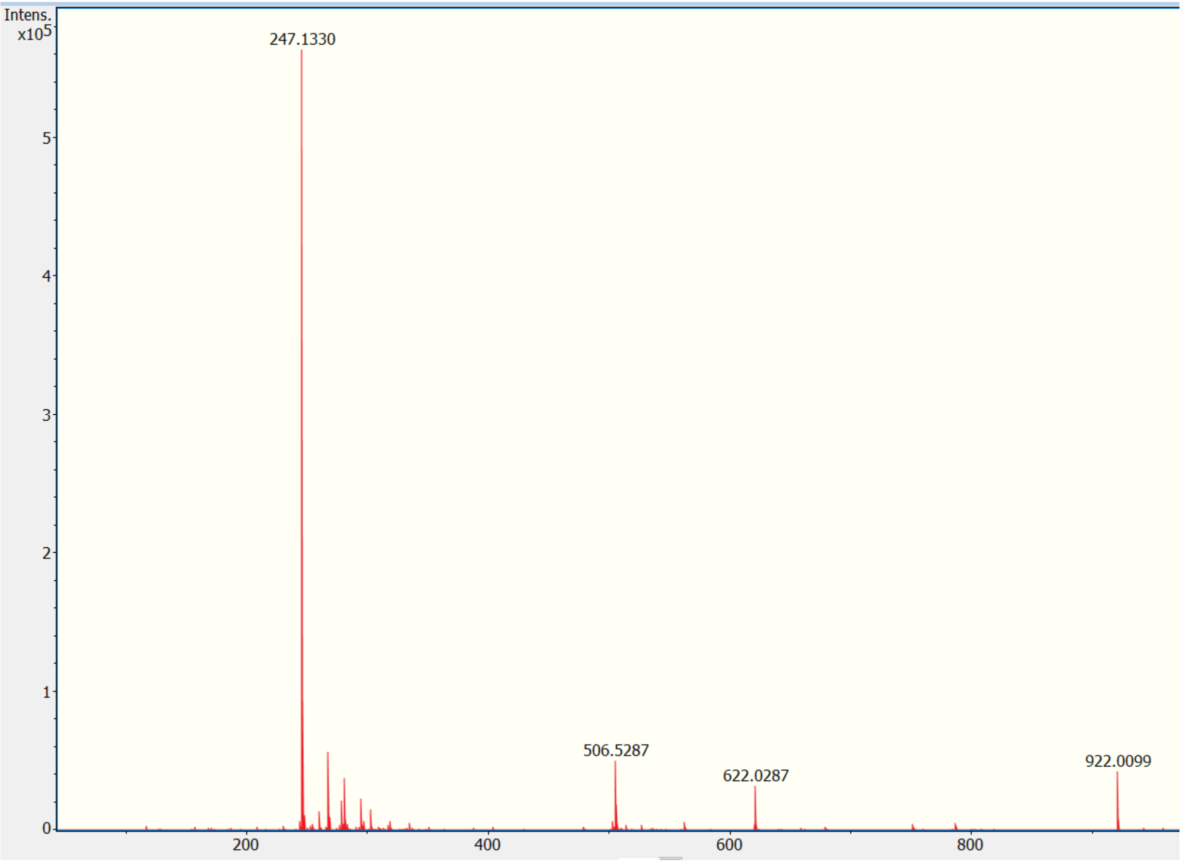
Intens.
x10^5
247.1330
506.5287
622.0287
922.0099

4,4-diethyl-5,8-dihydroxy-6-methylnaphthalen-1(4H)-one **(Compound 7)**

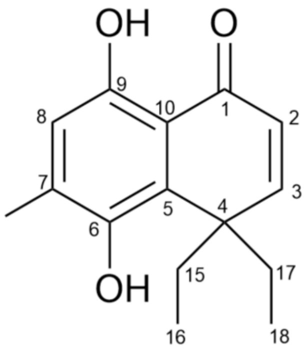

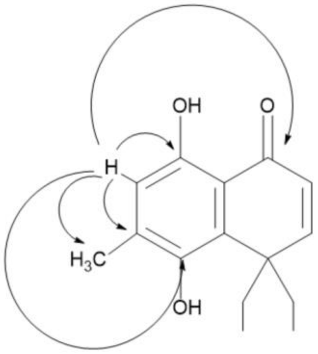

	desplazamiento 1H	desplazamiento 13C	acoplamiento
1	-	191.7	-
2	6,38	128.4	C-3; C-4; C-10
3	6,55	159.4	C-1; C-2; C-4; C-5; CH2-(15,17)
4	-	48.7	-
CH2-(15,17)	1.51(m), 2.71(m)	30.6	C-2; C-3; C-4; C-5; Me-(16,18)
Me-(16,18)	0.47	9.4	
5	-	130.6	-
6	-	143.8	-
7	-	130.0	-
8	6,66	117.2	C-1; C-6; C-9; C-10; Me-7
9	-	156.0	-
10	-	116.0	-
Me-7	2,22	16.9	C-6; C-7; C-8
OH-6	4,40	-	C-5; C-6
OH-9	12.71	-	C-7; C-8; C-9

1H

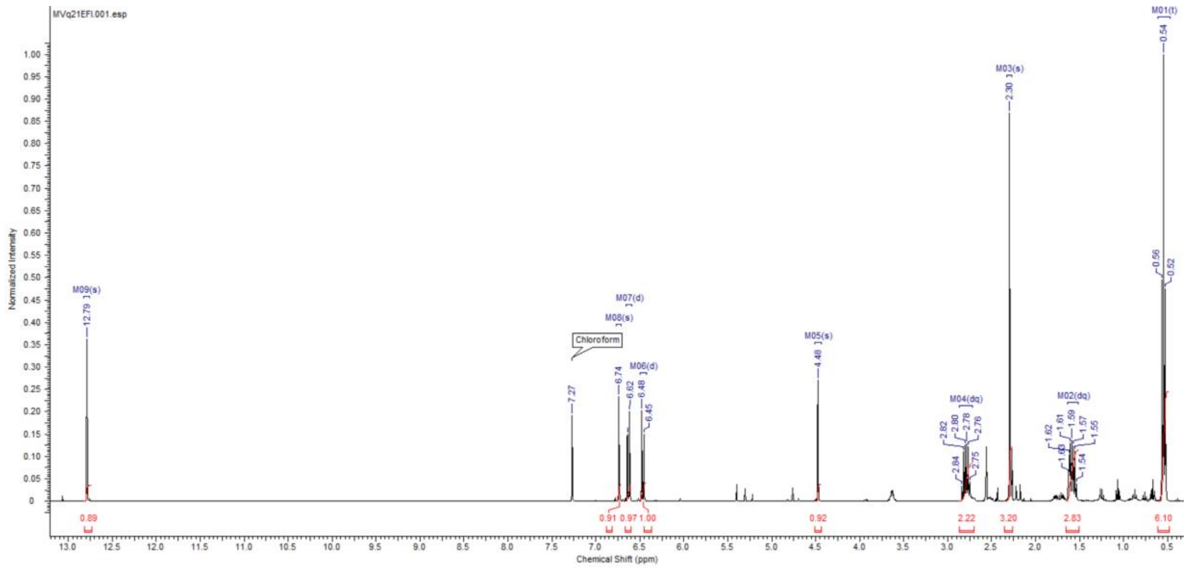
MVq21EFI.001.esp
Normalized Intensity
Chemical Shift (ppm)
Chloroform
M09(s)
12.79
M08(s)
M07(d)
M06(d)
7.27
6.74
6.62
6.48
6.45
M05(s)
4.48
M04(dq)
M03(s)
2.30
M02(dq)
M01(t)
0.54
0.56
0.52
0.89
0.91
0.97
1.00
0.92
2.22
3.20
2.83
6.10

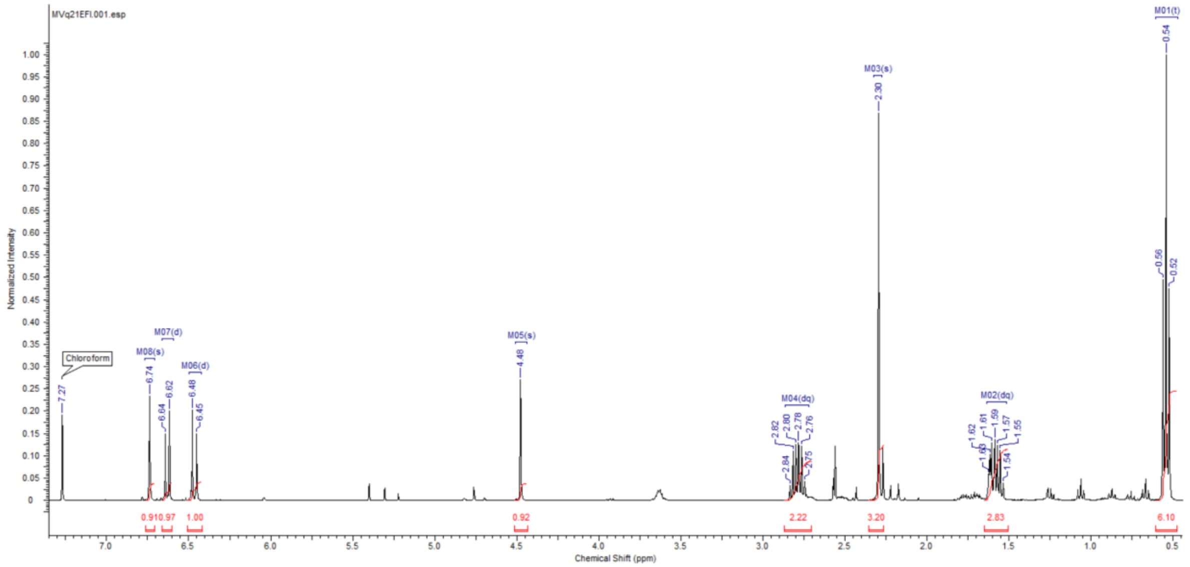
MVq21EFI.001.esp
Normalized Intensity
Chemical Shift (ppm)
Chloroform
7.27
M08(s)
M07(d)
M06(d)
6.74
6.64
6.62
6.48
6.45
M05(s)
4.48
M04(dq)
M03(s)
2.30
M02(dq)
M01(t)
0.54
0.56
0.52
0.91
0.97
1.00
0.92
2.22
3.20
2.83
6.10

13C

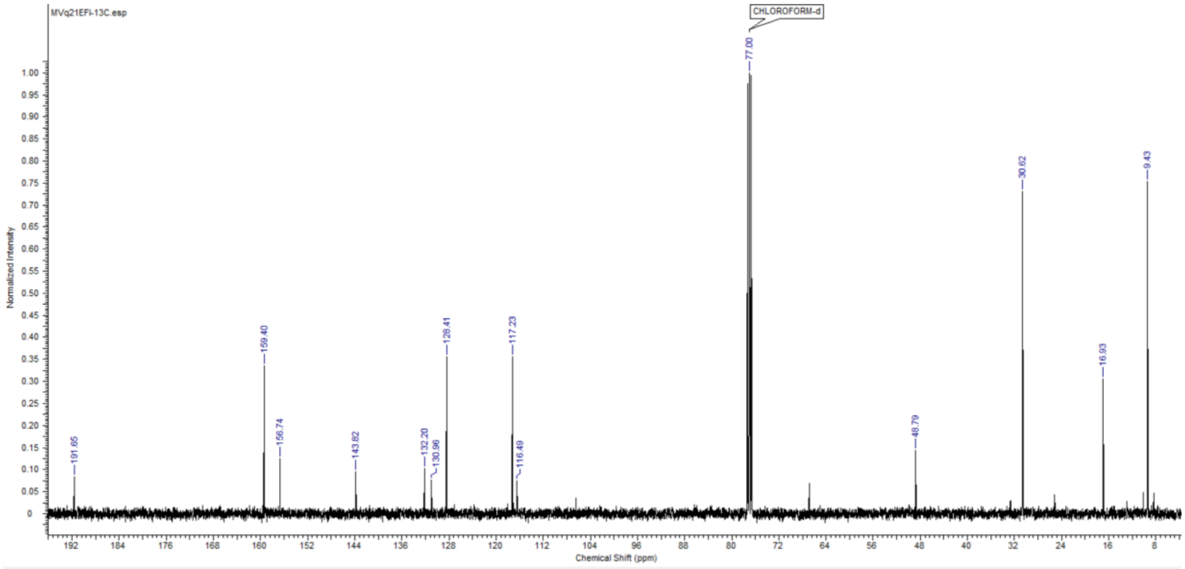
MVq21EFI-13C.esp
CHLOROFORM-d
77.00
159.40
156.74
191.65
143.82
132.20
130.96
128.41
117.23
116.49
48.79
30.62
16.93
9.43
Normalized Intensity
Chemical Shift (ppm)

HSQC

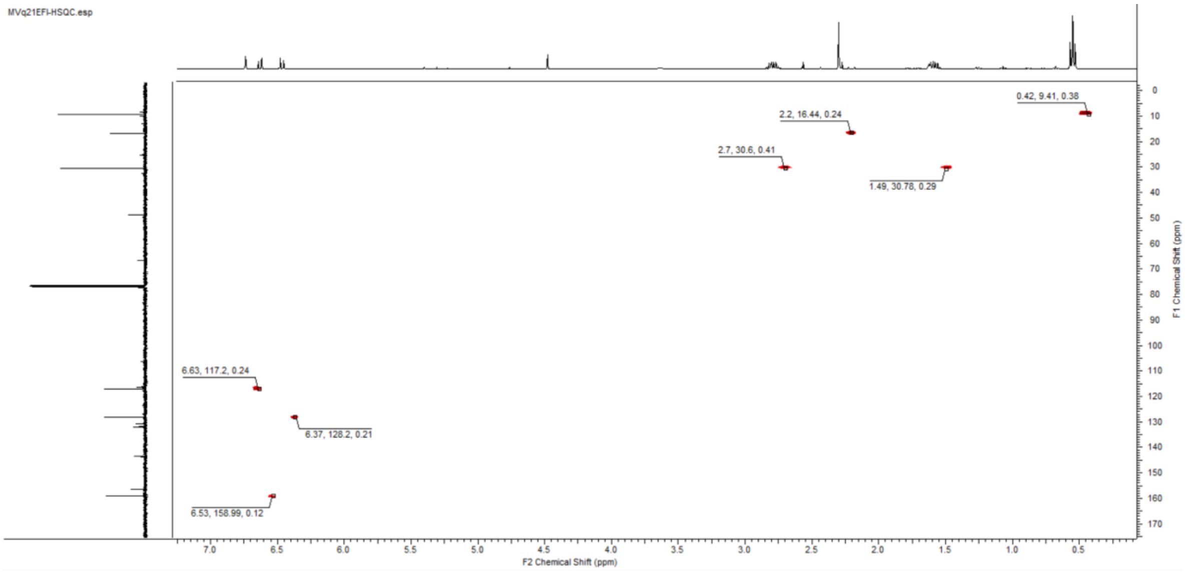
MVq21EFI-HSQC.esp
0.42, 9.41, 0.38
2.2, 16.44, 0.24
2.7, 30.6, 0.41
1.49, 30.78, 0.29
6.63, 117.2, 0.24
6.37, 128.2, 0.21
6.53, 158.99, 0.12
F2 Chemical Shift (ppm)
F1 Chemical Shift (ppm)

HMBC

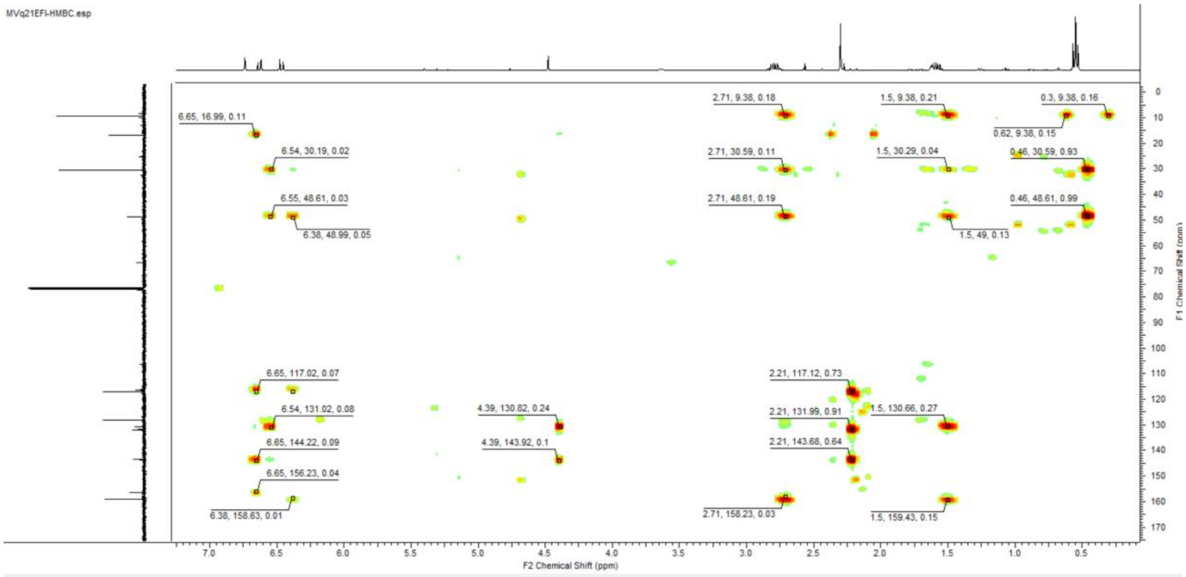

HRMS

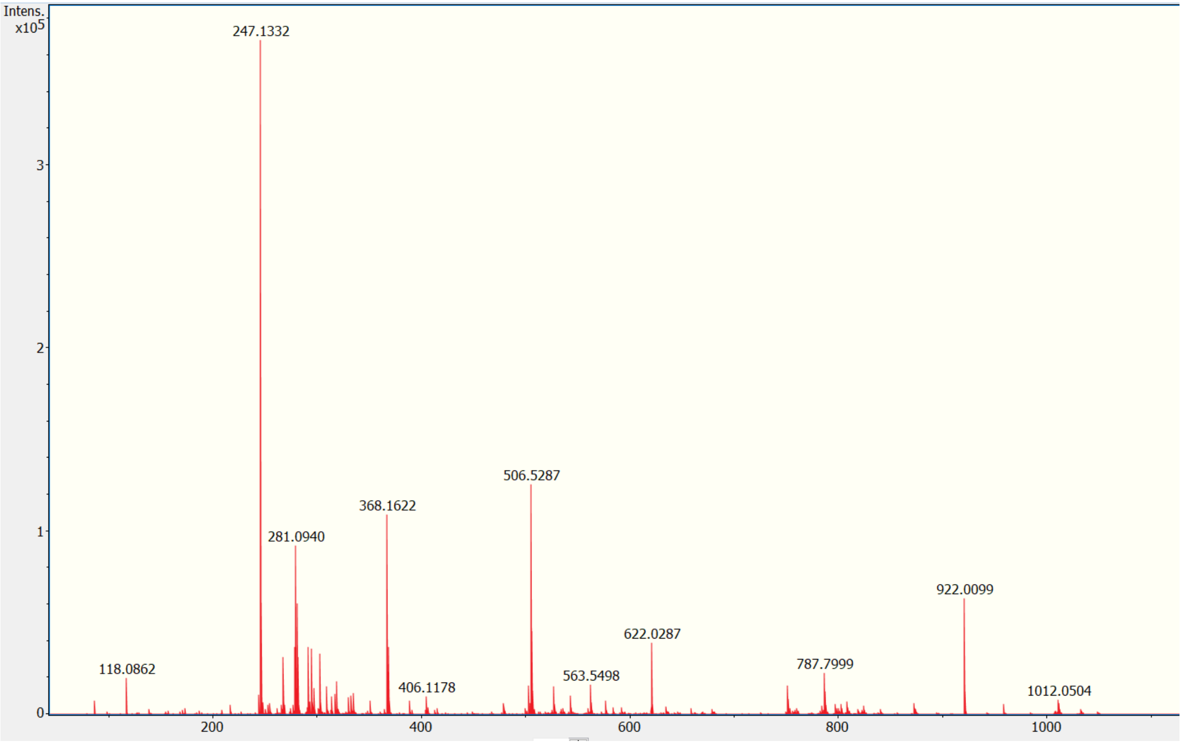
Intens.
x10^5
247.1332
506.5287
368.1622
281.0940
922.0099
622.0287
787.7999
118.0862
563.5498
406.1178
1012.0504

4,4-diethyl-5,8-dihydroxy-7-methylnaphthalen-1(4H)-one **(Compound 8)**

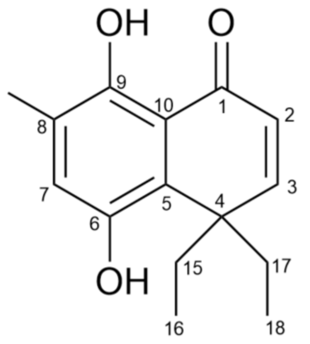

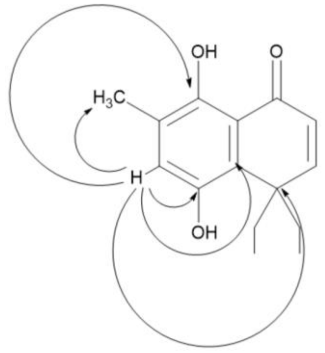

	desplazamiento 1H	desplazamiento 13C	acoplamiento
1	-	190,9	-
2	6,40	128.1	C-3; C-4; C-10
3	6,56	159.6	C-1; C-2; C-4; C-5; CH2-(15,17)
4	-	48.5	-
CH2-(15,17)	1.50(m), 2.68(m)	30.6	C-2; C-3; C-4; Me-(16,18)
Me-(16,18)	0.47	9.4	
5	-	128.3	-
6	-	144.2	-
7	-	124.8	-
8	6,69	125.2	C-4; C-5; C-6; C-9; Me-8
9	-	155.8	-
10	-	117.0	-
Me-8	2,15	15.2	C-8; C-9
OH-6	4,5	-	C-5; C-6
OH-9	13.00	-	C-8; C-9; C-10

1H

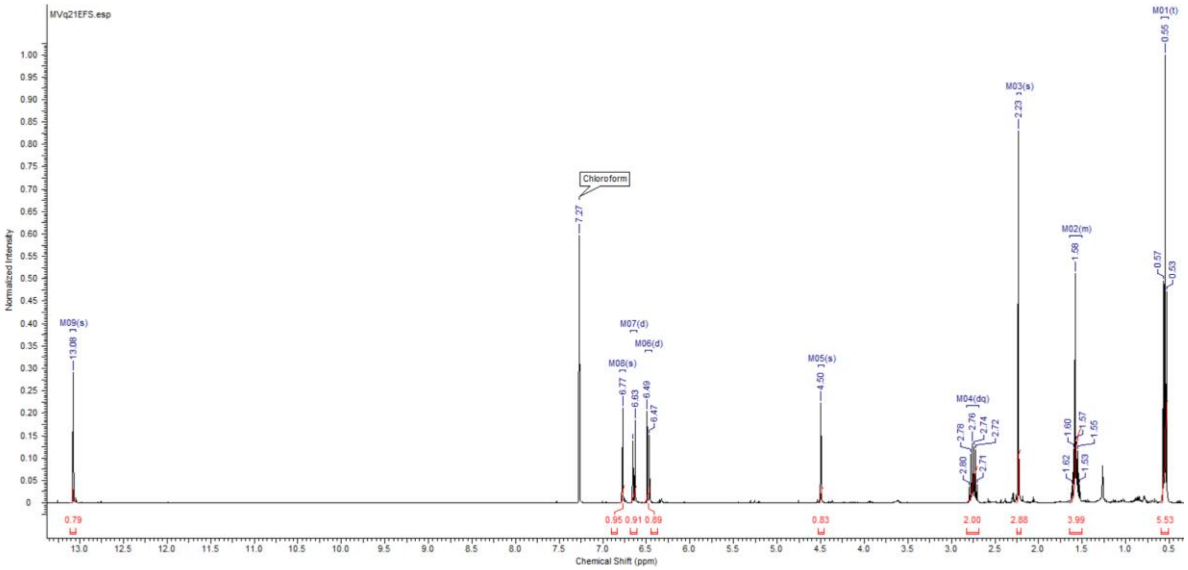
MVq21EFS.esp
Chloroform
M09(s)
M08(s)
M07(d)
M06(d)
M05(s)
M04(dq)
M03(s)
M02(m)
M01(t)
Normalized Intensity
Chemical Shift (ppm)

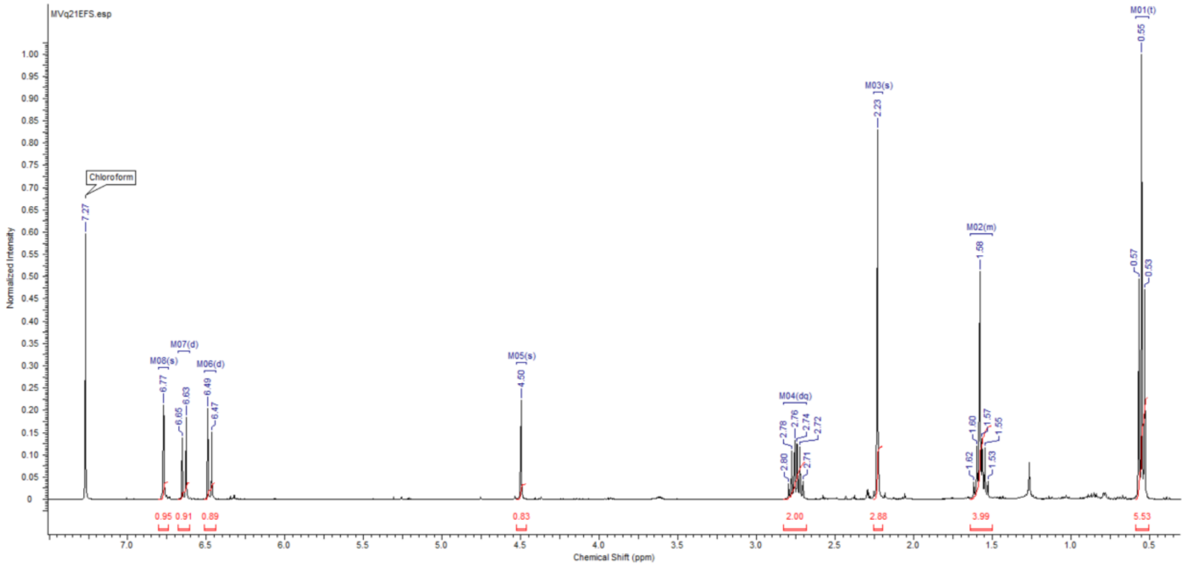
MVq21EFS.esp
Chloroform
M08(s)
M07(d)
M06(d)
M05(s)
M04(dq)
M03(s)
M02(m)
M01(t)
Normalized Intensity
Chemical Shift (ppm)

13C

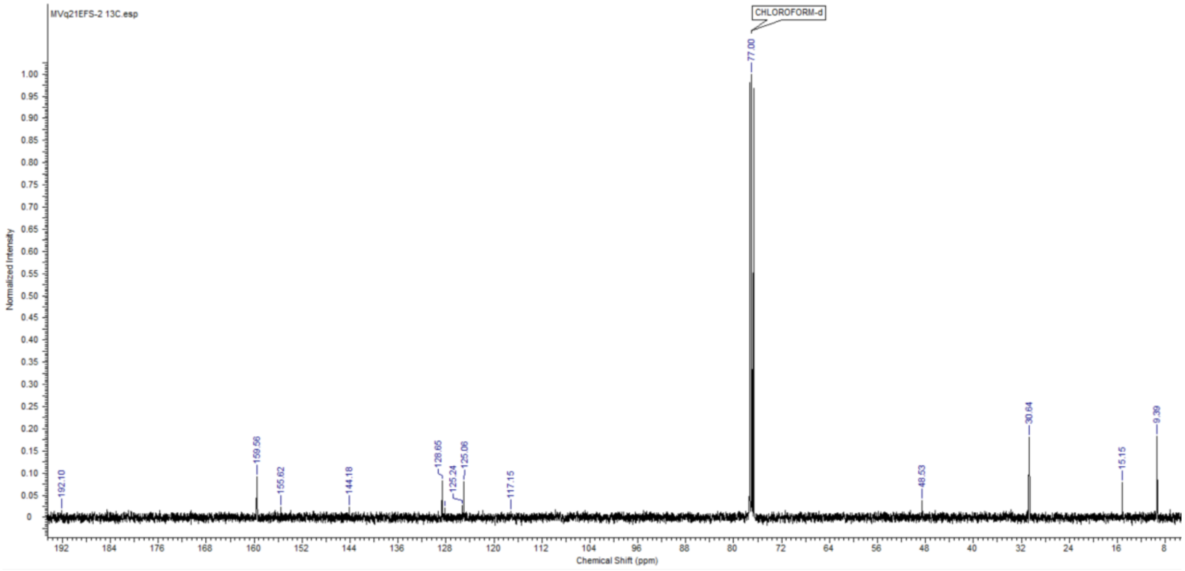
MVq21EFS-2 13C.esp
CHLOROFORM-d
77.00
192.10
159.56
155.62
144.18
128.65
125.24
125.06
117.15
48.53
30.64
15.15
9.39
Normalized Intensity
Chemical Shift (ppm)

HSQC

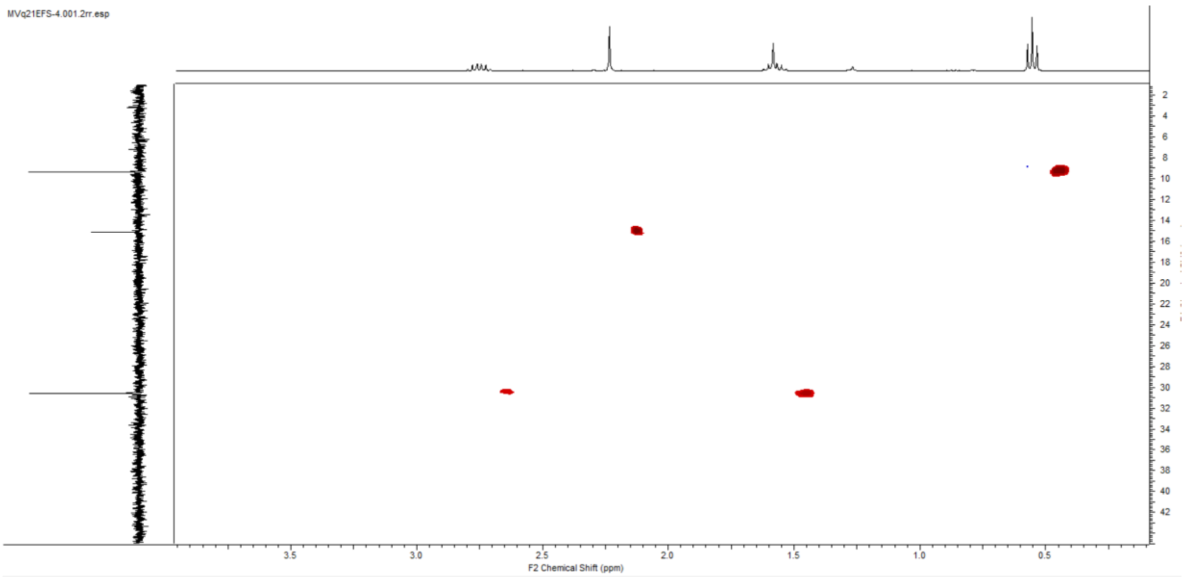
MVq21EFS-4.001.2rr.esp
F2 Chemical Shift (ppm)

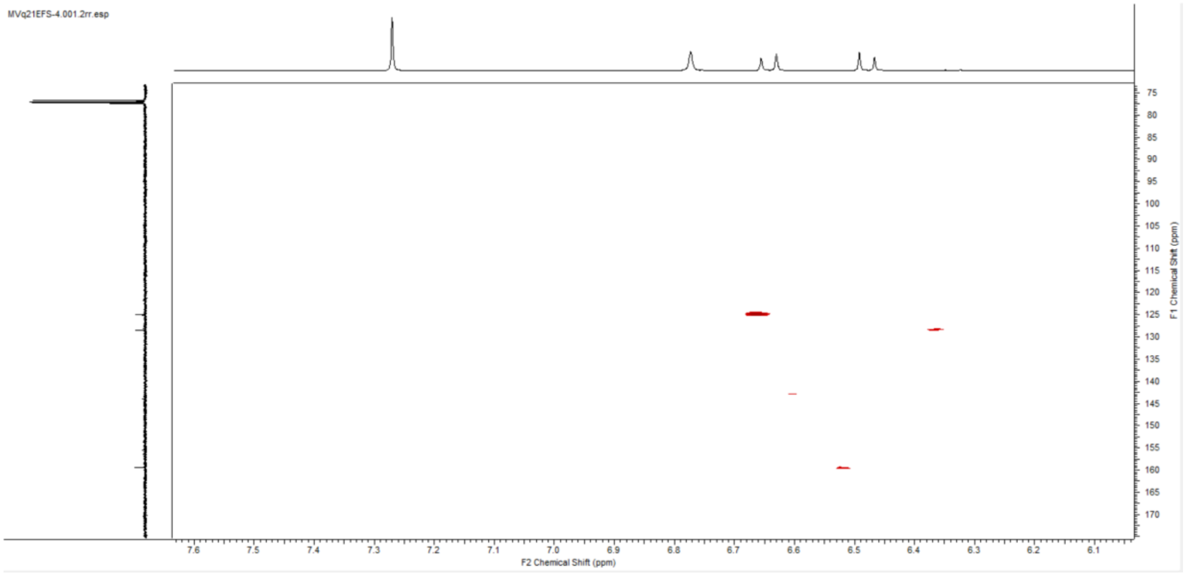
MVq21EFS-4.001.2rr.esp
F2 Chemical Shift (ppm)
F1 Chemical Shift (ppm)

HMBC

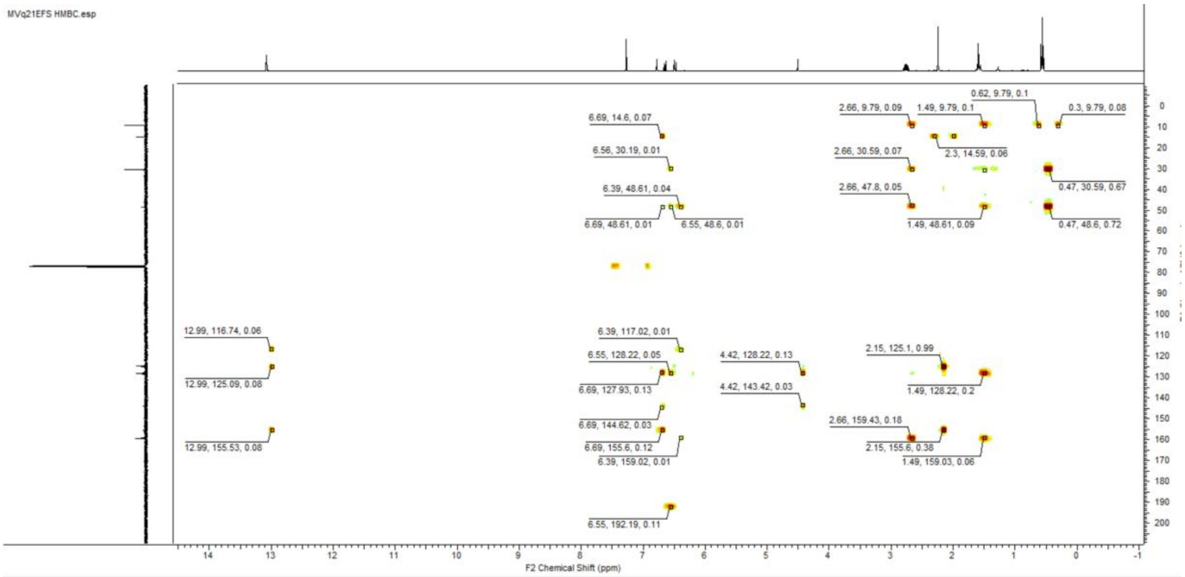
MVq21EFS HMBC.esp
6.69, 14.6, 0.07
6.56, 30.19, 0.01
6.39, 48.61, 0.04
6.69, 48.61, 0.01
6.55, 48.6, 0.01
2.66, 9.79, 0.09
1.49, 9.79, 0.1
0.62, 9.79, 0.1
0.3, 9.79, 0.08
2.3, 14.59, 0.06
2.66, 30.59, 0.07
0.47, 30.59, 0.67
2.66, 47.8, 0.05
1.49, 48.61, 0.09
0.47, 48.6, 0.72
12.99, 116.74, 0.06
12.99, 125.09, 0.08
12.99, 155.53, 0.08
6.39, 117.02, 0.01
6.55, 128.22, 0.05
6.69, 127.93, 0.13
6.69, 144.62, 0.03
6.69, 155.6, 0.12
6.39, 159.02, 0.01
6.55, 192.19, 0.11
4.42, 128.22, 0.13
4.42, 143.42, 0.03
2.15, 125.1, 0.99
1.49, 128.22, 0.2
2.66, 159.43, 0.18
2.15, 155.6, 0.38
1.49, 159.03, 0.06
F2 Chemical Shift (ppm)

HRMS

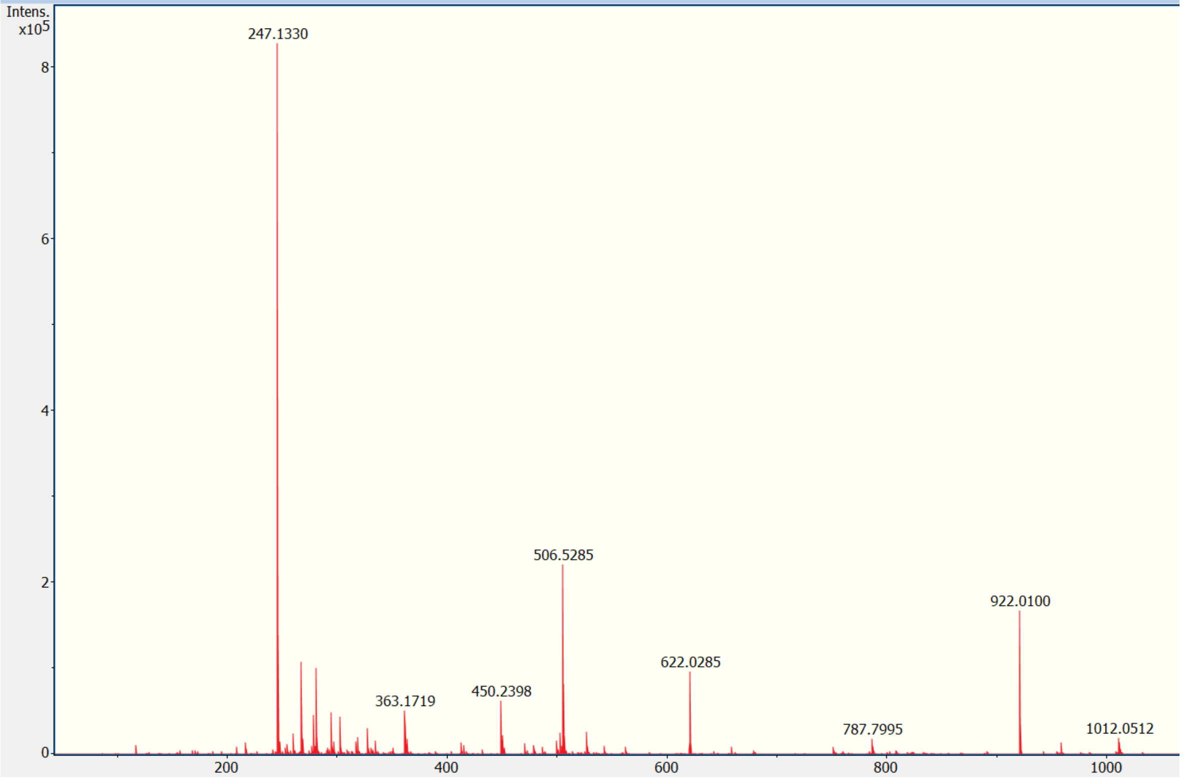

4,4-diethyl-5,8-dihydroxy-6,7-dimethylnaphthalen-1(4H)-one (Compound 9)

1H

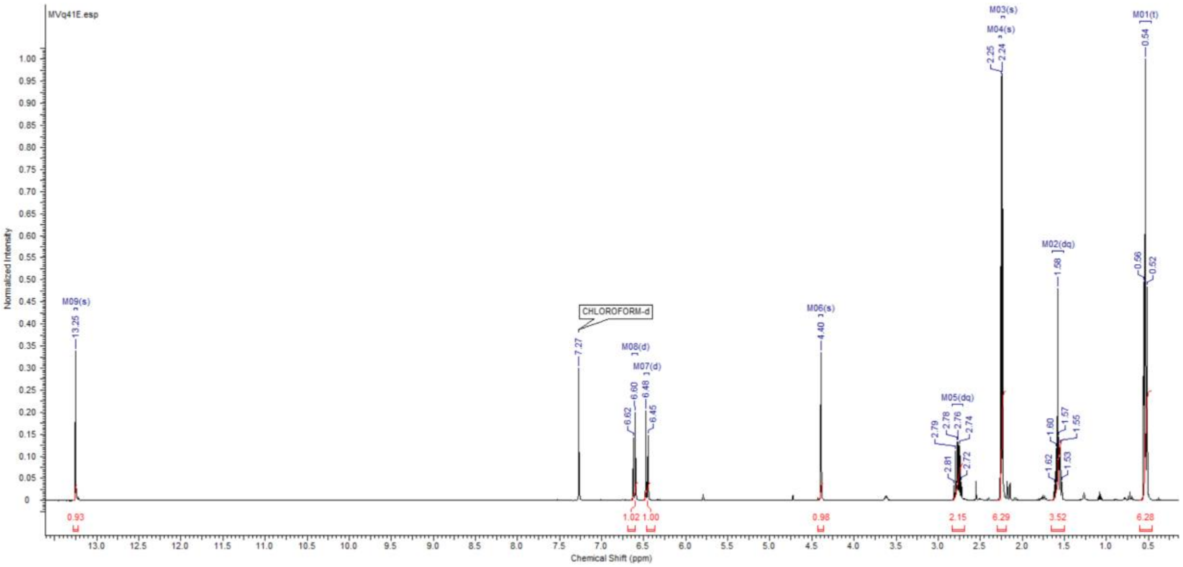

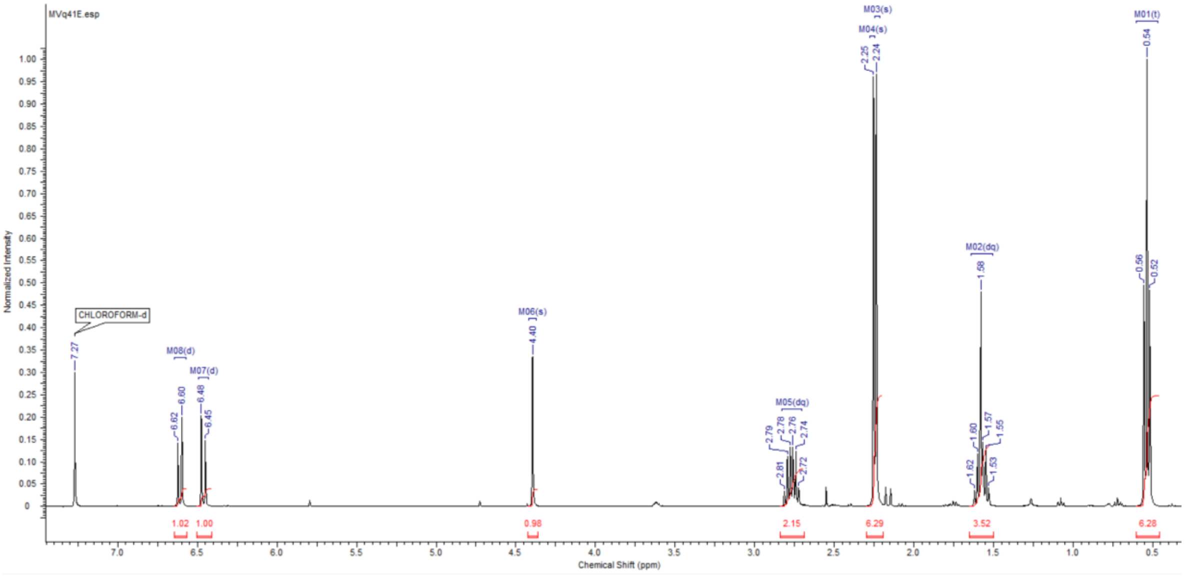
MVq41E.esp
CHLOROFORM-d
7.27
M08(d)
6.62
6.60
M07(d)
6.48
6.45
M06(s)
4.40
M05(dq)
2.81
2.79
2.78
2.76
2.74
2.72
M03(s)
M04(s)
2.25
2.24
M02(dq)
1.62
1.60
1.58
1.57
1.55
1.53
M01(t)
0.56
0.54
0.52
1.02
1.00
0.98
2.15
6.29
3.52
6.28
Normalized Intensity
Chemical Shift (ppm)

13C

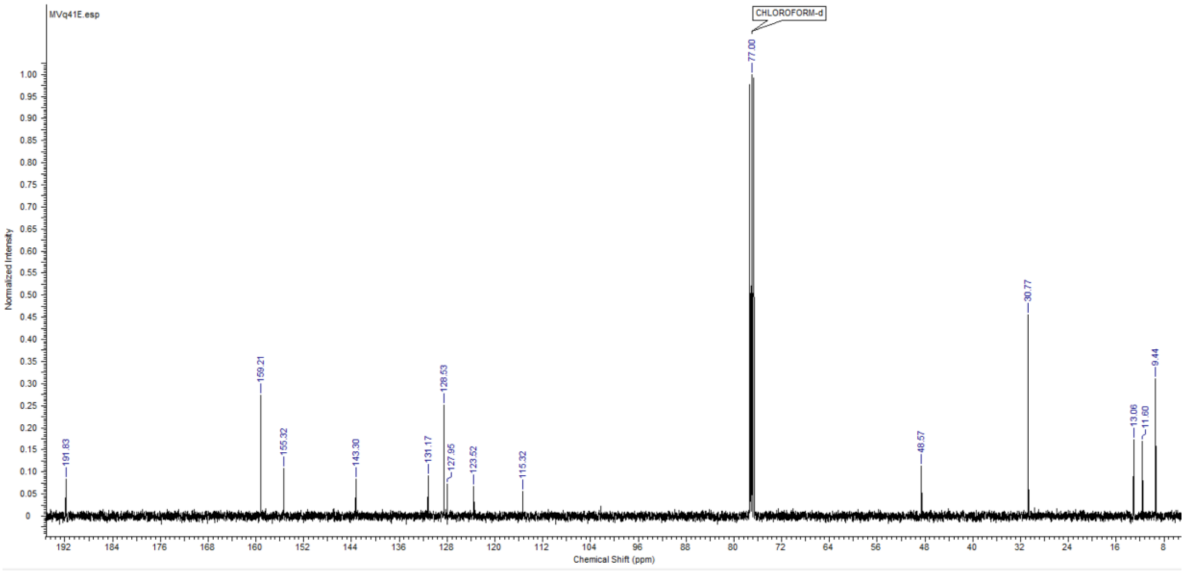
MVq41E.esp
CHLOROFORM-d
77.00
191.83
159.21
155.32
143.30
131.17
128.53
127.95
123.52
115.32
48.57
30.77
13.06
11.60
9.44
Normalized Intensity
Chemical Shift (ppm)

HRMS

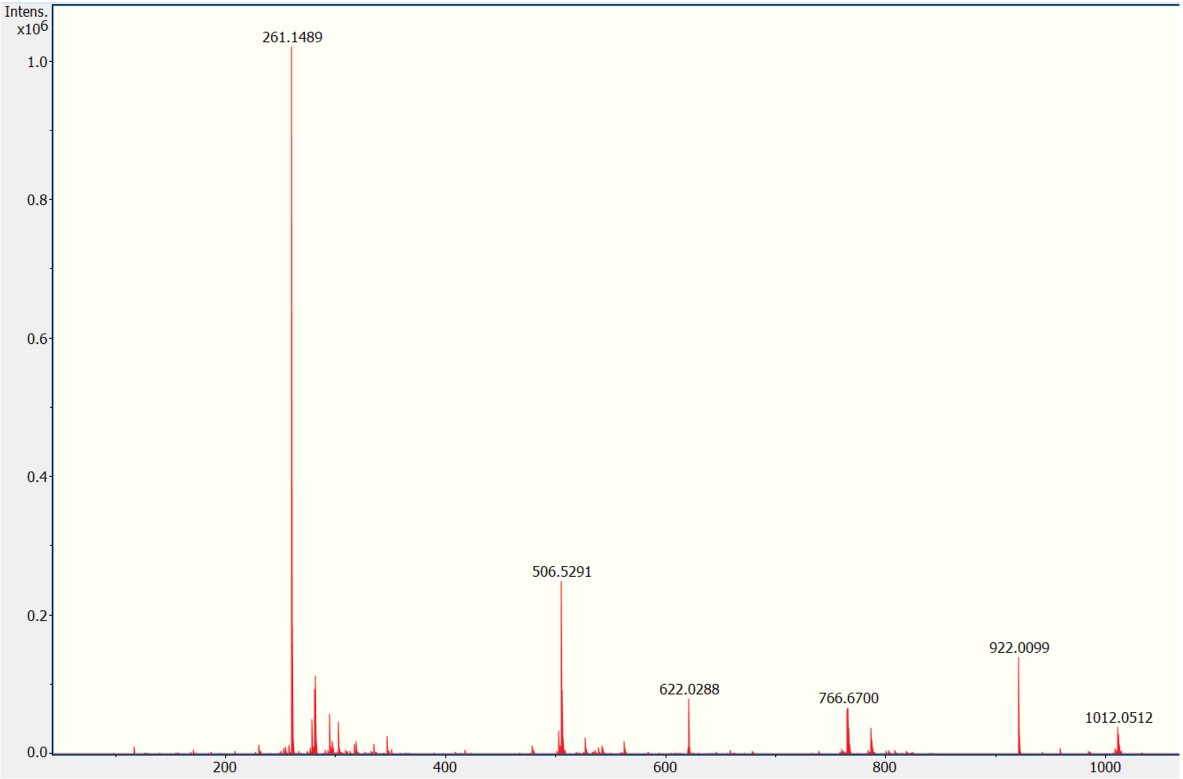

4,4-diethyl-5,8-dihydroxy-2,6,7-trimethylnaphthalen-1(4H)-one **(Compound 10)**

1H

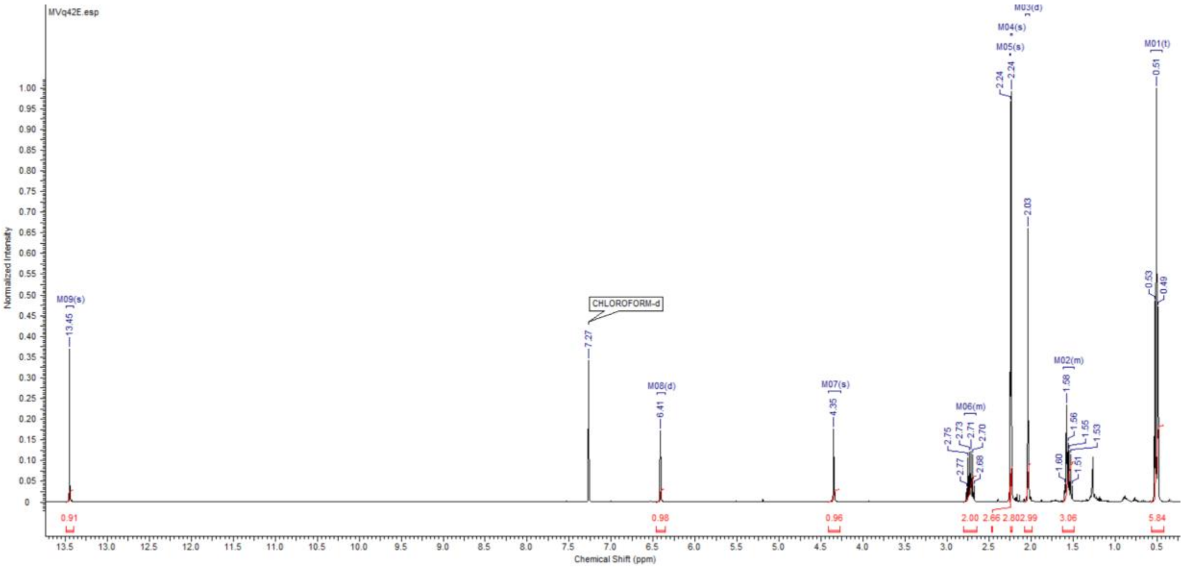

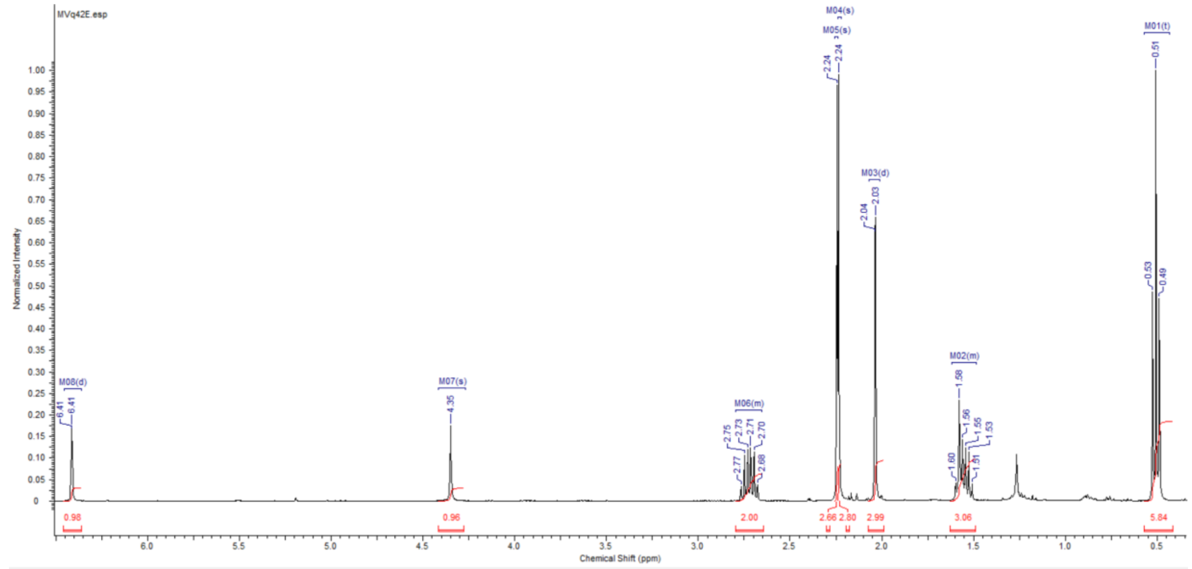
MVq42E.esp
M08(d)
M07(s)
M06(m)
M05(s)
M04(s)
M03(d)
M02(m)
M01(t)
Normalized Intensity
Chemical Shift (ppm)

13C

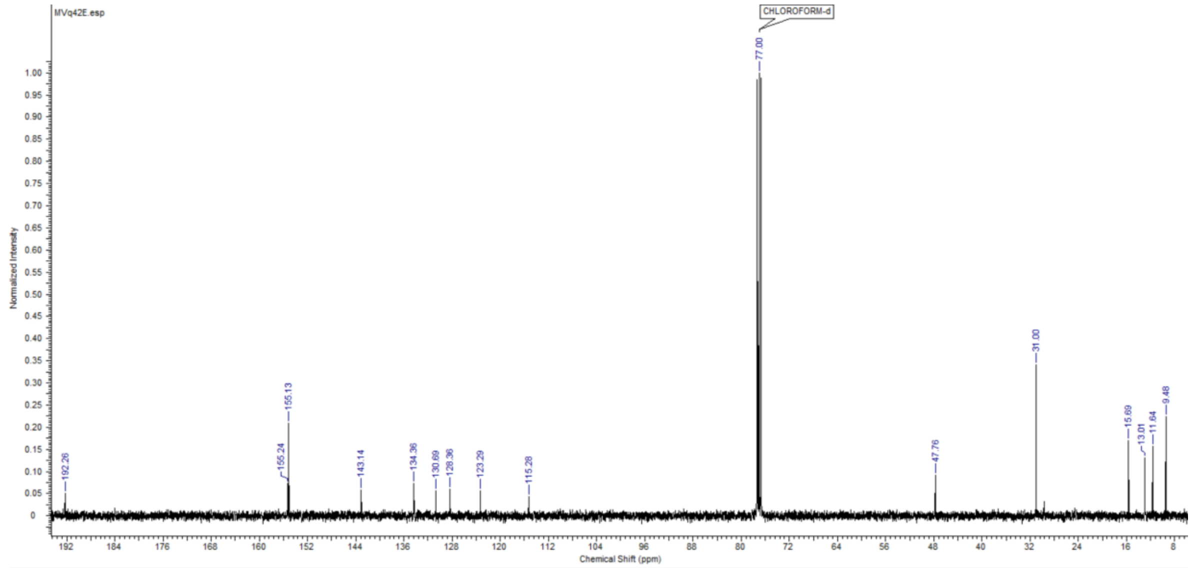
MVq42E.esp
CHLOROFORM-d
Normalized Intensity
Chemical Shift (ppm)

HRMS

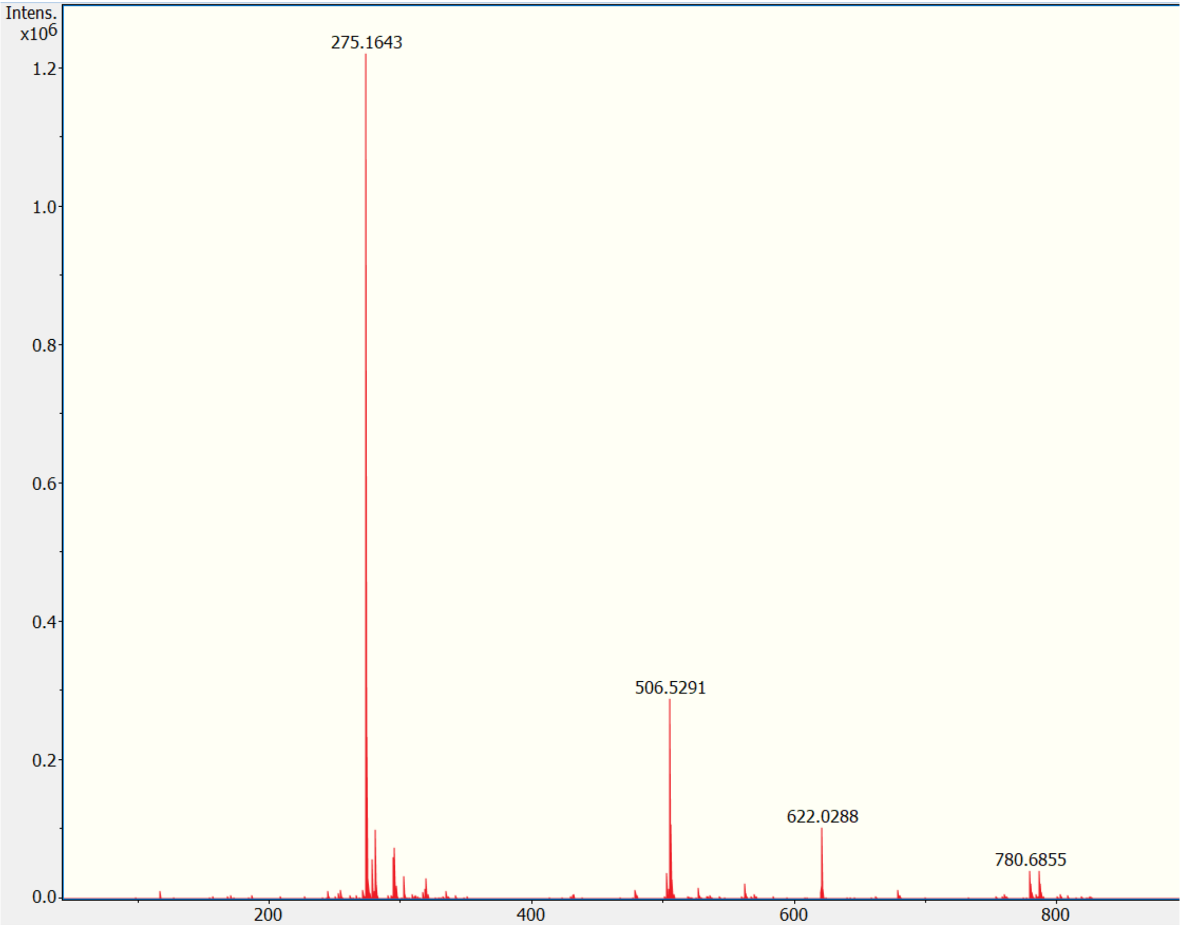

Intens.
x10^6
1.2
1.0
0.8
0.6
0.4
0.2
0.0
275.1643
506.5291
622.0288
780.6855
200
400
600
800
